# Reductive Dechlorination of Chlorinated Ethenes at the Sulfidated Zero-Valent Iron Surface: A Mechanistic DFT Study

**DOI:** 10.1021/acs.jpcc.4c00865

**Published:** 2024-02-28

**Authors:** Miroslav Brumovský, Daniel Tunega

**Affiliations:** University of Natural Resources and Life Sciences, Vienna, Department of Forest- and Soil Sciences, Institute of Soil Research, Peter-Jordan-Straße 82, 1190 Vienna, Austria

## Abstract

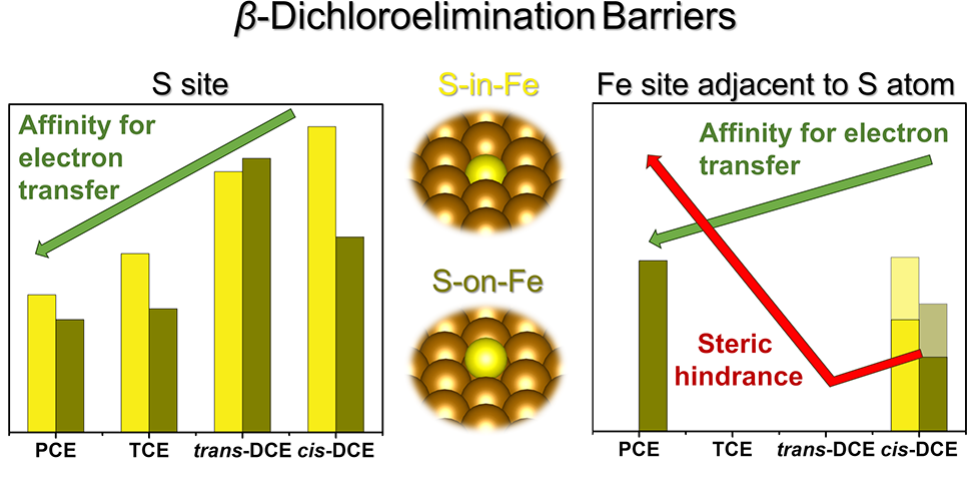

Sulfidated nano- and microscale zero-valent iron (S-(n)ZVI) has shown enhanced selectivity and reactive lifetime in the degradation of chlorinated ethenes (CEs) compared to pristine (n)ZVI. However, varying effects of sulfidation on the dechlorination rates of structurally similar CEs have been reported, with the underlying mechanisms remaining poorly understood. In this study, we investigated the *β*-dichloroelimination reactions of tetrachloroethene (PCE), trichloroethene (TCE), *cis*-1,2-dichloroethene (*cis*-DCE), and *trans*-1,2-dichloroethene (*trans*-DCE) at the S and Fe sites of several S-(n)ZVI surface models by using density functional theory. Dechlorination reactions were both kinetically and thermodynamically more favorable at Fe sites compared to S sites, indicating that maintaining the accessibility of reactive Fe sites is crucial for achieving high S-(n)ZVI reactivity with contaminants. At Fe sites adjacent to S atoms, the reactivity for CE dechlorination followed the order *trans*-DCE ≈ TCE > *cis*-DCE > PCE. PCE degradation was hindered at these sites due to the steric effects of S atoms. At the S sites, the energy barriers correlated with the CEs’ energy of the lowest unoccupied molecular orbital in the order PCE < TCE < DCE isomers. Our findings reveal that the experimentally observed selectivity of S-(n)ZVI materials for individual CEs can be explained by an interplay of the varying reactivities of Fe and S sites in CE dechlorination reactions.

## 1 Introduction

Sulfidation gained widespread acceptance as a simple, cheap, and environmentally acceptable approach to enhance the reactive lifetime and selectivity of nano- and microscale zero-valent iron ((n)ZVI) particles for in situ groundwater remediation.^1−3^ The high selectivity of sulfidated (n)ZVI (S-(n)ZVI) for target contaminants was attributed primarily to its increased hydrophobicity and lower reactivity with water compared to the pristine (n)ZVI, leading to the preferential sorption of hydrophobic contaminants at the S-(n)ZVI surface while simultaneously hindering particle corrosion.^4−8^

Chlorinated ethenes (CEs) such as trichloroethene (TCE), tetrachloroethene (PCE), and their less-chlorinated transformation products are pervasive groundwater contaminants.^9^ Although CEs represent some of the main contaminants treated by Fe-based materials in practice, the effect of sulfidation on their dechlorination mechanism is not completely understood.^2,10−12^ To date, dozens of studies consistently documented a remarkable increase in TCE dechlorination rate by S-(n)ZVI,^1,2^ but only a few works have addressed the reactivity of S-(n)ZVI with other CEs.^10−15^ While the reported enhancements in the dechlorination rates of these CEs are not fully consistent, likely as a result of different sulfidation procedures adopted in various studies, resulting in differences in the structure and composition of used S-(n)ZVI, a discernible trend can be observed: sulfidation significantly enhances the dechlorination of *trans*-1,2-dichloro-ethene (*trans*-DCE)^10−12^ but shows smaller improvements in the dechlorination of PCE and *cis*-1,2-dichloroethene (*cis*-DCE).^10−13^ Some studies even indicated inhibition of their degradation following (n)ZVI sulfidation.^13−15^ In addition, the dechlorination rates of individual CEs showed different trends in relation to the (n)ZVI sulfidation extent.^11,12^

The reductive dechlorination of CEs at (n)ZVI and iron sulfide (FeS_*x*_) surfaces proceeds by two major pathways: (i) *β*-dichloroelimination, consisting of the cleavage of two Cl atoms from adjacent C atoms, and (ii) hydrogenolysis, involving a replacement of a Cl atom by hydrogen.^16−19^ While the former occurs through a direct electron transfer at the Fe(S_*x*_) surface, the latter may, in principle, proceed through both electron transfer accompanied by a subsequent protonation or by a reductive reaction with adsorbed atomic hydrogen or hydride.^20−22^ The majority of past studies suggested that *β*-elimination is the dominant CE reduction pathway in S-(n)ZVI systems.^1,4,11,23−26^ This conclusion was based on several observed phenomena: (i) sulfidation promotes the accumulation of acetylene as a dechlorination product, while the formation of less-chlorinated intermediates is suppressed,^4,24,26,27^ (ii) FeS_*x*_ phases on the S-(n)ZVI surface hinder the adsorption of atomic hydrogen,^5,28,29^ implying its lower availability for hydrogenation and hydrogenolysis reactions, and (iii) FeS_*x*_ phases have a lower resistance to electron transfer than iron (oxyhydr)oxides that are typically present on the surface of pristine (n)ZVI exposed to water.^5,24,28^ Nevertheless, the S-induced reactivity enhancements in CE dechlorination were found to not correlate with CE reduction potentials (*E*^0^) or with energies of the lowest unoccupied molecular orbital (*E*_LUMO_) as would be expected for an electron transfer-controlled process.^10−12,15^ This discrepancy was attributed to specific S-(n)ZVI characteristics, such as the distribution and crystallinity of iron sulfide (FeS_*x*_) phases on the surface of S-(n)ZVI particles, the presence of metal impurities, and/or specific surface interactions at Fe/FeS_*x*_ sites, which may favor reactions with particular CEs.^11,13−15^ Several studies also suggested that the indirect reduction by adsorbed atomic hydrogen (H*_ads_) contributes to the dechlorination of CEs, even representing the dominant dechlorination pathway for less-chlorinated CEs at a low S surface coverage.^10,12,30^ The factors governing S-(n)ZVI reactivity with CEs in the studies described above have been inferred only from indirect observations and correlations between the dechlorination rates and various physicochemical properties of CEs. A deeper mechanistic understanding of CE dehalogenation reactions via both *β*-elimination and hydrogenolysis pathways at the S-(n)ZVI surface, as well as the role of sulfur in the active sites, is still lacking.

In our recent study, we shed more light on the fundamental effects of iron sulfidation on the *β*-dichloroelimination of TCE.^29^ In particular, we showed that the pristine Fe surface is extremely reactive toward TCE dechlorination. Contrarily to assumptions made in the prior literature,^31^ we demonstrated that S atoms intrinsically inhibit contaminant reduction by hindering the electron transfer toward molecules adsorbed at the S sites. Such a poisoning effect is well-known in the transition-metal catalysis.^32−35^ Our findings further showed that the overall promoting effect of sulfidation on the reactivity of (n)ZVI materials with contaminants is indirect, primarily consisting of protecting the (n)ZVI surface from corrosion and passivation^4−8^ and thereby conserving the reactive Fe surface sites.

Herein, we aimed to explore whether such intrinsic inhibitory effects on electron-transfer-controlled dechlorination reactions can shed more light on the varying reactivity enhancements observed for different CEs after (n)ZVI sulfidation. Reaction energy profiles of *β*-dichloroelimination of PCE, TCE, *cis*-DCE, and *trans*-DCE at various sites of the pristine and S-doped Fe(110) surfaces were calculated using density functional theory (DFT) methods. To assess the overall feasibility of dechlorination at the investigated sites, reactions with cleaved Cl atoms at both *cis* and *trans* positions for PCE and TCE were modeled. This study helps to understand the mechanisms governing the selectivity of S-(n)ZVI for individual CEs.

## 2 Computational Details

### 2.1 Methods

All electronic structure calculations were performed using the spin-polarized plane-wave DFT method implemented in the Vienna ab initio Simulation Package (VASP).^36−38^ The interactions between the valence and core electrons were treated with the projector-augmented wave framework,^39,40^ and the electronic exchange–correlation was described using the generalized gradient approximation (GGA) with the Perdew–Burke–Ernzerhof (PBE) functional.^41^ The kinetic energy cutoff for plane waves in all calculations was 400 eV. Long-range dispersion forces were accounted for using the DFT-D3 approach with the Becke-Johnson damping.^42,43^ The Brillouin zone integrations were performed on a 2 × 2 × 1 Monkhorst–Pack^44^*k*-point grid for all surface structures. The convergence condition for the electronic self-consistent cycle was 10^–6^ eV. Structural relaxations were performed using the conjugate gradient algorithm as implemented in VASP until the residual forces on all atoms were less than 0.01 eV/Å. The climbing image nudged elastic band method (CI-NEB)^45^ was used to accurately describe the minimum energy paths, including transition states. The effect of solvation was included in the calculations using the implicit solvation model VASPsol^46,47^ on the gas-phase optimized geometries. Details on the calculations of adsorption energies and reaction profiles are provided in Text S1 in the Supporting Information.

### 2.2 Modeled Systems

To investigate the effect of surface sulfidation on the Fe reactivity with CEs, we used the following four surface slab models: (i) pristine Fe(110) surface slab represented by three Fe planes with the lateral dimensions 15.881 × 13.889 Å, (ii) the Fe(110) slab with one surface Fe atom replaced by an S atom (further referred to as “S-in-Fe(110)”), (iii) a model with one S atom bridging two Fe atoms at the hollow site of the Fe(110) surface (further referred to as “S-on-Fe(110)”), and (iv) the Fe(110) surface doped with several S atoms on the hollow sites in a regular fashion, representing 1/8 monolayer coverage (termed as “S_1/8 ML_-Fe(110)”). The Fe(110) facet represents the thermodynamically most stable surface of pristine *α*-Fe.^48^ The S-in-Fe(110) and S-on-Fe(110) models were used in previous studies to reveal the suppressing effect of S on the adsorption of water and atomic hydrogen^6,28,49^ as well as in our recent study to showcase the intrinsic inhibition of TCE *β*-elimination at the S site.^29^ The regularly sulfidated Fe(110) surface was used to validate the effects of S atoms observed on Fe surfaces doped with a single S atom. The 1/8 monolayer coverage represents low S coverage,^29^ at which both S and Fe sites are reasonably accessible for contaminants. All surface models contained a 25 Å-thick vacuum layer in the direction perpendicular to the surface to decouple adjacent slabs. Models were allowed to fully relax during CE adsorption and CI-NEB calculations with constant lattice parameters, except for the S_1/8 ML_-Fe(110) surface, in which two bottom Fe layers were fixed to prevent deformation of the slab caused by steric repulsion between the S atoms and the adsorbed CE molecules. The energy differences between the constrained and fully unconstrained adsorption complexes were negligible (typically <3 kJ mol^–1^).

## 3 Results and Discussion

### 3.1 Dechlorination of CEs at the Pristine Fe(110) Surface Proceeds with Negligible Barriers

To provide a reference for calculations at the S-doped surfaces, it is crucial to understand the reactivity of CEs on the pristine Fe(110) surface. The reductive dechlorination of CEs involves three consecutive steps: (a) adsorption of the CE molecule onto the surface, (b) surface-mediated reduction reactions, and (c) desorption of dechlorinated products from the surface.^16,50,51^ We have shown in our recent studies that the pristine Fe(110) surface is extremely reactive with CEs.^15,29^ PCE exhibited stable (nondissociated) physisorption and chemisorption at the Fe(110) surface, with virtually no barrier for the transition between the two states (Figure S1). Unconstrained structural relaxations of TCE and *cis*-DCE adsorption complexes led directly to chemisorption accompanied by a spontaneous cleavage of two C–Cl bonds from adjacent C atoms.^15^

To fully quantify the *β*-dichloroelimination reaction barriers for all CEs with ≥2 Cl atoms in their molecules at the pristine Fe(110) surface, CI-NEB calculations were performed. Except for PCE, the CE adsorption complexes were optimized with fixed C–Cl bond lengths using the GADGET code.^52^ These chemisorption complexes were used as the initial structures in the CI-NEB calculations.

In the chemisorbed state, all CEs adsorbed via the C=C bond at the atop Fe site, with a distance of ∼2 Å between the C atoms and the Fe site (Figure S2). Compared with their planar gas phase geometry, their structure was deformed in the chemisorbed complexes. The length of the C=C bond notably increased by ∼0.1 Å, and the dihedral angles decreased from 180 to ∼130° (Table S1), indicating a strong activation for subsequent dechlorination reactions.^15,16,50,51,53^ The adsorption energies in the solvent ranged from −171.6 to −206.3 kJ mol^–1^ (Table S2), with the most favorable adsorption calculated for the chemisorbed PCE.

The CI-NEB calculations revealed negligible or even no barriers for the *β*-dichloroelimination reactions of CEs at the pristine Fe(110) surface, including both *cis* and *trans* reaction pathways for PCE and TCE (Figure S3), indicating that the activation energies for these reactions are negligible. The CE dissociation upon contact with pristine Fe has been experimentally documented with Auger electron spectroscopy, temperature-programmed desorption, and photoelectron spectroscopic methods under ultrahigh vacuum.^54,55^ These results indicate that pristine Fe(110) exhibits extremely high reactivity with CEs when its surface is not covered by corrosion products.

The virtual absence of CE dechlorination barriers at the pristine Fe(110) surface prevented us from reliably predicting trends in the reactivity of various CEs at this surface. However, experimental studies indicated that the CE dechlorination rates with (n)ZVI materials inversely correlate with the energy of the lowest unoccupied molecular orbital (*E*_LUMO_),^11,15^ resulting in faster removal of CEs with higher degrees of chlorination.^56^ This trend was also predicted in previous DFT calculations, where all but one dissociating Cl atom in the molecule were kept frozen.^51^ These observations suggest that dechlorination rates of CEs at the pristine Fe(110) surface are predominantly governed by an electron-transfer process,^19^ in particular *β*-dichloroelimination.

### 3.2 At the S-in-Fe Site, Barriers for CE Reductive Dechlorination Correlate with *E*_LUMO_, with a Preference for the *trans*-*β*-Elimination Pathway

To explore the intrinsic effects of incorporating sulfur on the reductive dechlorination of CEs, we first employed an Fe(110) surface model with one Fe atom substituted by one S atom, termed S-in-Fe(110). This structure has been previously suggested as a simplified representation of S-nZVI particles prepared using the cosulfidation (“one-pot”) method.^6,49^

Upon structural relaxations, all CEs formed stable adsorption complexes at the S-in-Fe(110) site, with their molecules horizontally oriented to the slab surface and the C=C bond positioned 3.1–3.4 Å above the S atom (Figure S4). The geometry of CE molecules in their adsorption complexes showed only slight distortions compared with the gas phase (Table S1). Notably, no significant elongation of C=C or C–Cl bonds was observed, suggesting a lower activation for dechlorination reactions compared to the Fe site on the pristine Fe(110) surface. The adsorption energies ranged from −71.3 to −102.1 kJ mol^–1^, with the weakest interaction observed for *trans*-DCE, followed by *cis*-DCE, TCE, and PCE (Table S2). The calculated CE adsorption energies at the S-in-Fe(110) site were approximately half of the strength of CE adsorption at the pristine Fe(110) surface. The weaker CE interaction with S sites compared to Fe sites was also evident from the pure DFT energy contribution to the total adsorption energy (Table S2). While DFT contributed about 50% at the Fe(110) surface, the CE adsorption energy at the S-in-Fe(110) site was predominantly attributed to the dispersion forces, indicating a much lower electronic interaction at the S sites compared to the Fe sites, as previously reported for TCE in our recent study.^29^

**Figure 1:**
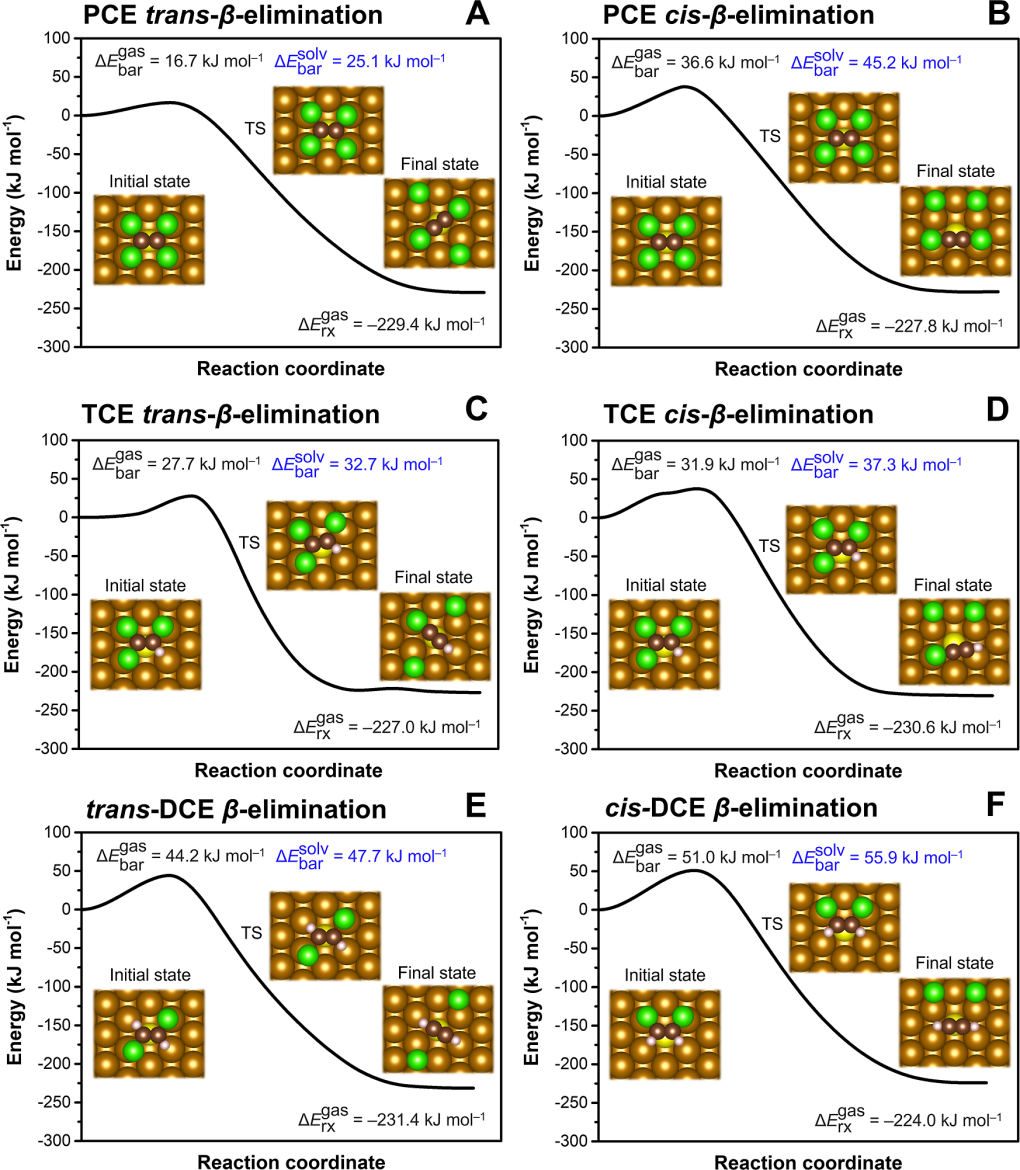
Reaction profiles of chloroethene *β*-dichloroelimination reactions at the S-in-Fe(110) site: (A,B) PCE, (C,D) TCE, (E) *trans*-DCE, and (F) *cis*-DCE. CI-NEB calculations were performed in the gas phase (values in black). The solvent effect on the reaction barrier was included using a continuum solvation model with the structures of reactants and transition states taken from the CI-NEB calculation (values in blue). TS denotes the transition state.

The optimized adsorption complexes served as the initial structures for calculating dechlorination barriers. While dechlorination reactions of CEs are expected to proceed via sequential one-electron-transfer reactions,^19^ the structural relaxations of dechlorination intermediates with one cleaved C–Cl bond resulted in the spontaneous cleavage of a second Cl atom from a vicinal C atom. Therefore, we calculated reaction barriers for complete *β*-elimination reactions, including both *cis* and *trans* pathways for PCE and TCE (Figure 1). CE dechlorination at the S-in-Fe(110) site showed higher reaction barriers compared to the pristine Fe(110) surface, consistent with our recent study on TCE reactivity at sulfidated Fe surfaces.^29^ The solvent-corrected *β*-elimination barriers ranged from 25.1 to 55.9 kJ mol^–1^, indicating that CE reduction is still feasible at these sites, although not as favorable as at the pristine Fe surface. The inclusion of solvation and correction for zero-point vibrational energy (ZPE) had only a small effect on the energy barriers as they typically canceled each other (Figure S5A).

The calculated reaction profiles at the flat S-in-Fe(110) site reveal two significant trends: (i) *β*-dichloroelimination becomes more favorable with increasing chlorination degree of CEs and (ii) *trans*-*β*-dichloroelimination is more favorable than *cis*-*β*-dichloroelimination. These trends align with previous observations for reactions with unmodified (n)ZVI particles.^21,56^ The reaction barriers for *trans*-*β*-dichloroelimination reactions show a strong correlation with the *E*_LUMO_ values of CEs (Figure S5B), as would be expected for an electron-transfer-controlled process.^16,57^ Altogether, the incorporation of an S atom to the Fe surface intrinsically hinders CE dechlorination but does not appear to alter the reactivity trends in electron-transfer-mediated reactions among various CEs compared to (n)ZVI.

Note that the dispersion forces, which predominantly control the interaction between the S sites and adsorbates, were treated here using the empirical D3 correction with Becke-Johnson damping.^42,43^ Depending on the studied system, the empirical D3 dispersion correction could overestimate or underestimate the interaction energies.^58−60^ Therefore, benchmark calculations were performed on the reactant and transition state structures for the most favorable dechlorination pathways using the *meta*-GGA strongly constrained and appropriately normed (SCAN) functional, which inherently accounts for short- and medium-range dispersion forces.^61^ Remarkably, the SCAN-calculated reaction barriers at the S-in-Fe(110) sites showed excellent agreement with the values obtained using the PBE+D3 approach (Figure S6), confirming the high accuracy of the PBE+D3 results.

### 3.3 CE Dechlorination at the S-on-Fe Site Occurs Preferentially via the *cis-β*-Elimination Pathway

Understanding the impact of different S site architectures on contaminant dechlorination pathways is essential for gaining insights into the role of sulfur in the reactivity of S-(n)ZVI. Therefore, we investigated the intrinsic effects of sulfur on CE adsorption and dechlorination also using an Fe(110) surface model with one S atom adsorbed at an Fe hollow site (termed “S-on-Fe(110)”). This surface model has been previously used in computational studies as a simplified representation of S-(n)ZVI surface prepared by sulfidation of presynthesized (n)ZVI particles (i.e., “postsulfidation” method).^6,49^

At the S-on-Fe(110) site, similar to the S-in-Fe(110) site, all CEs formed stable adsorption complexes (Figure S7). The adsorbed CE molecules exhibited a tilted configuration above the S atom, with C atoms ∼3.4 Å far from the S sites. While the gas phase structures of the adsorbates were largely preserved (Table S1), the lengths of C–Cl bonds were slightly affected by their proximity to the slab: bonds oriented toward the slab were elongated by ∼0.02–0.04 Å, while bonds oriented away from the slab were shortened by ∼0.01–0.02 Å. The adsorption energies ranged from −51.5 to −73.0 kJ mol^–1^ and showed a similar trend as that at the S-in-Fe(110) site (Table S2). However, the adsorbate interactions at the S-on-Fe(110) site were less favorable compared to the S-in-Fe(110) site due to the smaller contact area between the adsorbed CE molecule and the free iron surface caused by the steric hindrance of the preadsorbed S atom.

**Figure 2:**
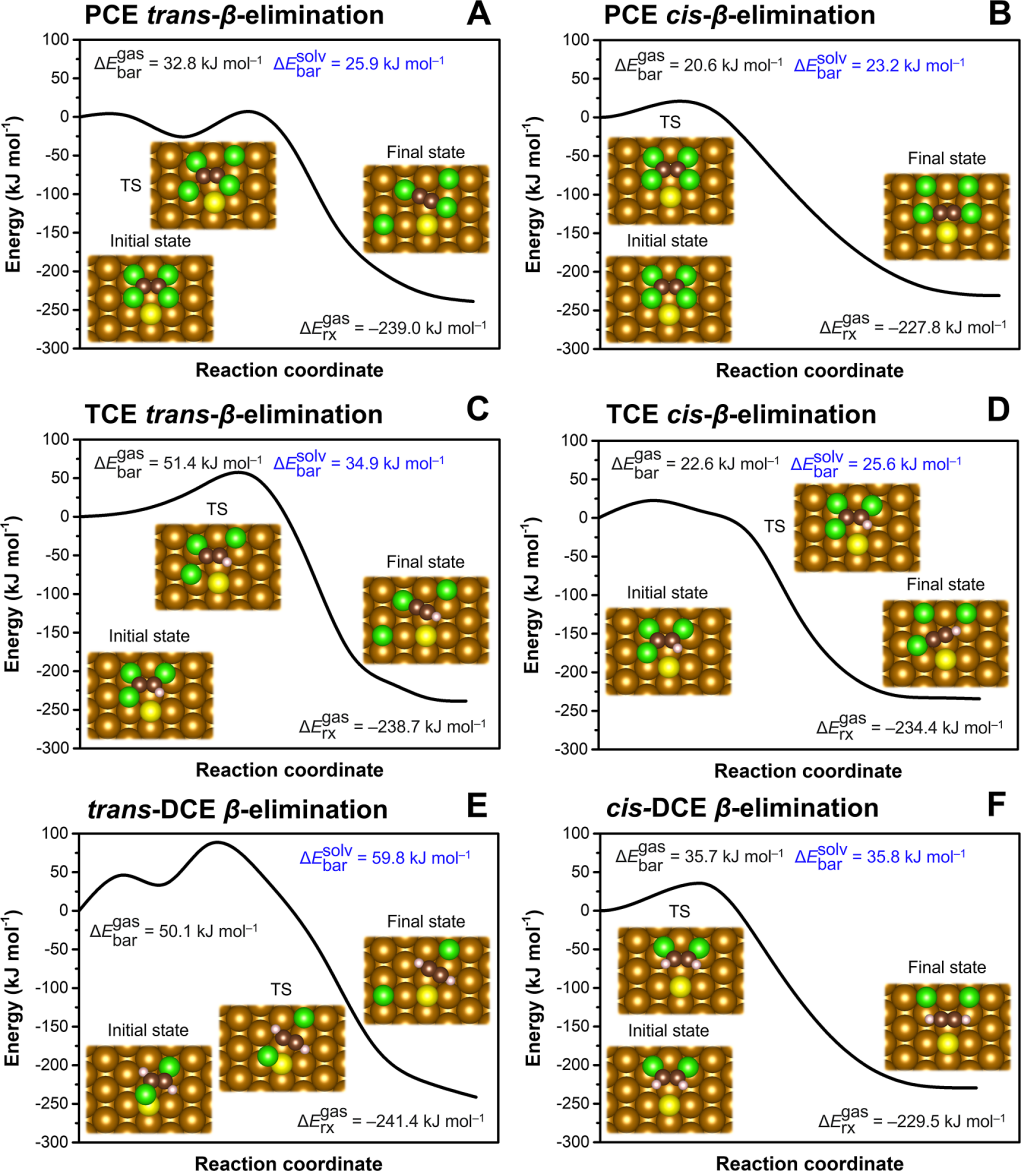
Reaction profiles of chloroethene *β*-dichloroelimination reactions at the S-on-Fe(110) site: (A,B) PCE, (C,D) TCE, (E) *trans*-DCE, and (F) *cis*-DCE. CI-NEB calculations were performed in the gas phase (values in black). The solvent effect on the reaction barrier was included using a continuum solvation model with the structures of reactants and transition states taken from the CI-NEB calculation (values in blue). TS denotes the transition state.

Using the relaxed adsorption complexes of CEs at the S-on-Fe(110) site as reactants, the *β*-dichloroelimination barriers were calculated, including both the *cis* and *trans* pathways for PCE and TCE (Figure 2). The calculated solvent-corrected barriers ranged from 23.2 to 59.8 kJ mol^–1^, being higher compared to those at the pristine Fe(110) surface but similar to those at the S-in-Fe(110) site discussed above. The inclusion of solvation and ZPE correction had only a minor impact on the calculated barriers (Figure S8A), except for the *trans*-*β*-dichloroelimination of TCE, where a shift of the transition state geometry occurred.

The calculated reaction profiles reveal that the *β*-dichloroelimination of CEs at the S-on-Fe(110) site is more favorable for compounds with a higher degree of chlorination and that the reaction barriers correlate with the *E*_LUMO_ of CEs (Figure S8B). While this trend has been consistently observed at both S-in-Fe(110) and S-on-Fe(110) sites, the sites showed different reaction stereoselectivities, with *cis*-*β*-dichloroelimination being more favorable at the S-on-Fe(110) site due to the tilted adsorption configurations of CEs (Figure S7). Consequently, the most pronounced differences between the two S site architectures were observed in the dechlorination reactions of *cis*- and *trans*-DCE. While the *β*-elimination of *trans*-DCE occurred at the S-in-Fe(110) site with a barrier 8.2 kJ mol^–1^ lower compared to that of the *cis*-isomer, it faced a higher barrier by 24.0 kJ mol^–1^ at the S-on-Fe(110) site. This suggests that increasing the abundance of S-on-Fe sites at the expense of S-in-Fe sites on the surface of S-(n)ZVI could potentially enhance the *cis*-DCE removal. However, the accessibility of reactive Fe sites likely plays a more important role than the S site architecture as discussed below.

The accuracy of the PBE+D3 results for the S-on-Fe(110) site was validated by using benchmark calculations of the reaction barriers for the most favorable dechlorination pathways for each CE using the SCAN functional. The SCAN-calculated values showed a similar trend as observed with the PBE+D3 approach, except for a slightly lower barrier for TCE compared to PCE (Figure S9). However, this discrepancy is small, as all calculated barriers fall within the range of 18–25 kJ mol^–1^, indicating that the PBE+D3 method is sufficiently accurate to infer reactivity trends of CEs, which are mostly controlled by dispersion forces at the S sites.

### 3.4 Incorporation of a Single S Atom Slightly Increases the Dechlorination Barrier for PCE and *cis*-DCE at Nearby Fe Sites

While sulfidation intrinsically hinders CE dechlorination, as shown above, it protects surface Fe sites adjacent to S atoms from corrosion by preventing the adsorption of water and H* at these sites.^6,29,49^ Consequently, these Fe sites are likely the primary active sites for the transfer of electrons to contaminants at the S-(n)ZVI surface. Therefore, the CE *β*-dichloroelimination pathways at the Fe sites adjacent to both S-in-Fe(110) and S-on-Fe(110) sites were also investigated. In the case of S-in-Fe(110), the nearest Fe atom to the S atom was considered, while for S-on-Fe(110), a more distant Fe atom was chosen to avoid steric repulsion between the S atom and the CE molecule.

Like at the pristine Fe(110) surface, structural relaxations of CE molecules over the Fe sites led to the spontaneous cleavage of two C–Cl bonds for most compounds, except for physisorbed PCE and chemisorbed *cis*-DCE. To perform calculations of dechlorination and chemisorption profiles, the adsorption complexes of chemisorbed PCE, TCE, and *trans*-DCE were optimized with constrained C–Cl bond lengths. These optimized structures served as initial and final states for the calculation of the dechlorination and chemisorption reaction profiles, respectively.

**Figure 3:**
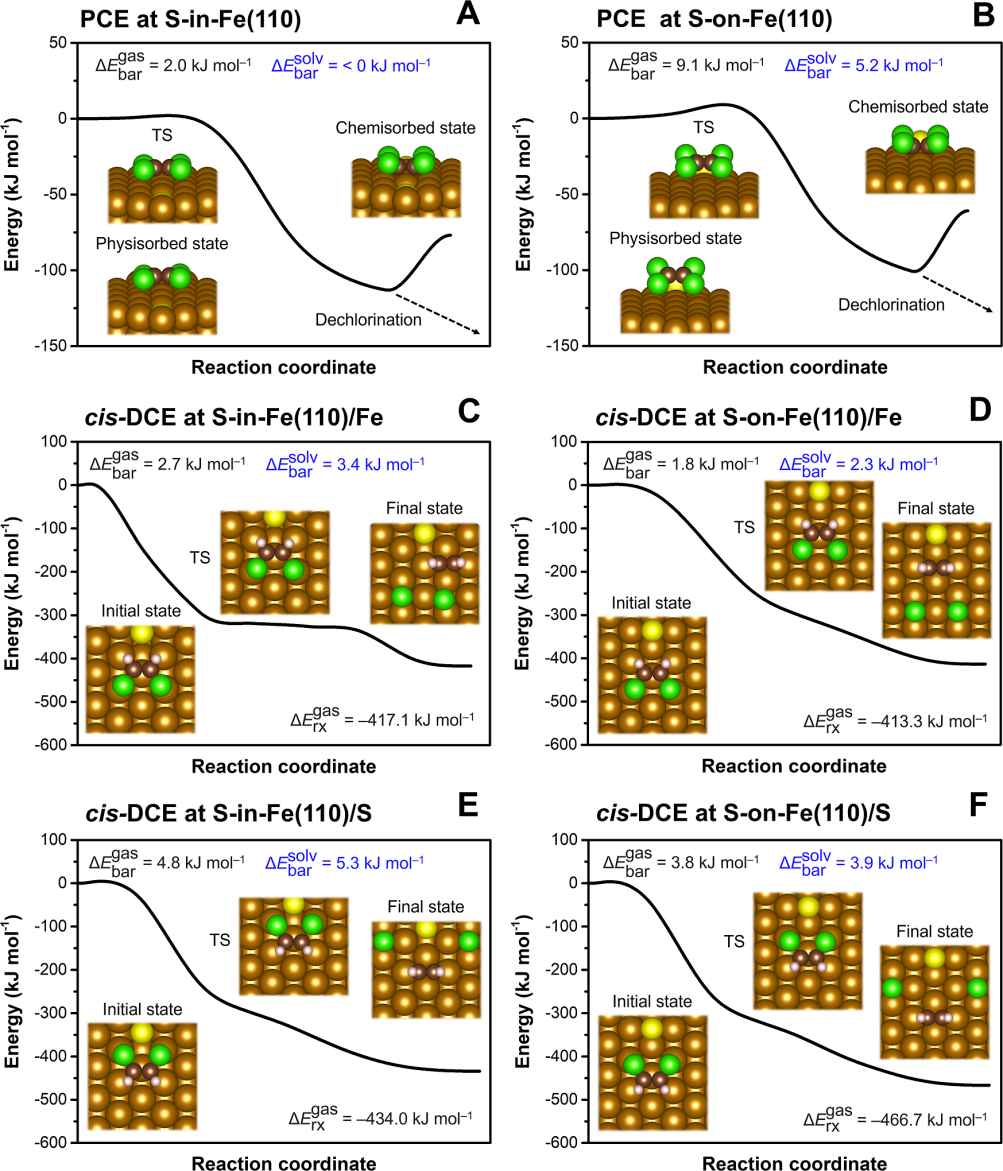
Effects of sulfidation on the reactivity of nearby Fe sites on the S-in-Fe(110) and S-on-Fe(110) surfaces. (A,B) Reaction profiles of PCE chemisorption. (C,D) *β*-Dichloroelimination profiles of *cis*-DCE initially adsorbed with Cl atoms toward the Fe slab. (E,F) *β*-Dichloroelimination profiles of *cis*-DCE initially adsorbed with Cl atoms toward the S atom. CI-NEB calculations were performed in the gas phase (values in black). The solvent effect on the reaction barrier was included using a continuum solvation model with the structures of reactants and transition states taken from the CI-NEB calculation (values in blue). TS denotes the transition state. The chemisorbed states near S atoms in panels A and (B) were calculated with fixed C–Cl distances to prevent spontaneous cleavage of Cl atoms during structural relaxations.

In the physisorbed states at both Fe sites, the PCE molecule was slightly tilted away from the S atom with minor changes in geometry compared to the gas phase, namely, elongated C–Cl bonds oriented toward the Fe slab, especially noticeable at the S-on-Fe(110) surface (Table S1). The transition from the physisorbed to the chemisorbed state at both Fe sites was accompanied by a small energy barrier, reaching 5.2 kJ mol^–1^ at the Fe site adjacent to the S-on-Fe(110) site and disappeared completely at the Fe site near the S-in-Fe(110) site after accounting for solvation effects (Figure 3A,B). The low stability of PCE chemisorption complexes at Fe sites near S atoms is further evident from the energy profiles, which favor PCE dechlorination after overcoming the initial barrier associated with the bending of the PCE molecule. This can be explained by the steric hindrance by the nearby S atom, which pushes chemisorbed PCE away from the Fe site, ultimately leading to its dechlorination.

CEs chemisorbed at the Fe sites exhibited structures similar to those observed at the pristine Fe(110) surface (Figures S10 and S11), with a distance of ∼2 Å between the C atoms and the Fe site, elongated C=C bonds, and distorted dihedral angles of ∼130° (Table S1). Despite the proximity of the S atom, the chemisorbed CE molecules displayed high activation toward dechlorination reactions, comparable to that at the pristine Fe(110) surface. The solvent-corrected adsorption energies ranged from −167.6 to −189.7 at the Fe sites of the S-in-Fe(110) surface and from −155.0 to −182.7 kJ mol^–1^ at the Fe sites of the S-on-Fe(110) surface (Table S2). The adsorption energies were only slightly lower than those calculated at the pristine Fe(110) surface, suggesting a minimal effect of the nearby S atoms on the interactions between Fe sites and CEs. Among all CEs, *cis*-DCE exhibited the most favorable adsorption energy due to the unconstrained relaxation of its adsorption complex. As *cis*-DCE can undergo only *cis*-*β*-dichloroelimination, its adsorption geometry was optimized at both Fe sites in two configurations, with the Cl atoms oriented (a) toward the Fe surface and (b) toward the S atom. These two configurations exhibited similar adsorption energies at both Fe sites, indicating that both adsorption complexes may represent reactants in the *cis*-DCE dechlorination reactions.

The strong adsorption of CE molecules (except PCE) at Fe sites adjacent to S atoms can be observed from the projected density of states (Figure S12). Compared to their physisorption complexes at the S site, the highest occupied molecular orbitals (π_C=C_) of the TCE, *cis*-DCE, and *trans*-DCE chemisorbed at the Fe site exhibited greater hybridization with the Fe 3d orbitals, resulting in larger shifts and broadening of their bands. The same trend was observed for the nonbonding π-orbitals of Cl. In the *cis*-DCE chemisorption complex, which has been relaxed with unconstrained C–Cl bond lengths, a weakening of the C–Cl and C–H bonds was also apparent, as documented by a shift of the corresponding orbitals to higher energies. This electronic analysis clearly shows that CE molecules are more activated for dechlorination reactions at Fe sites compared to S sites, provided that there are no steric barriers.

The CI-NEB calculations did not reveal any barriers for the *β*-dichloroelimination of chemisorbed PCE, TCE, and *trans*-DCE (Figure S13), indicating that the activation energies of these reactions are negligible. However, the *cis*-DCE dechlorination barriers slightly increased at both Fe sites compared to the pristine Fe(110) surface (Figure 3C–F). The solvation-corrected barriers reached 3.4 and 5.3 kJ mol^–1^ at the Fe sites adjacent to the S-in-Fe(110) site for *cis*-DCE adsorption configurations with the Cl atoms oriented toward the Fe surface and S atom, respectively. Adjacent to the S-on-Fe(110) site, the *β*-dichloroelimination barriers were 2.3 and 3.9 kJ mol^–1^, respectively. The increase in *cis*-DCE dechlorination barriers is consistent with the formation of a stable undissociated chemisorption complex during full structural relaxations in contrast to the pristine Fe(110) surface, where *cis*-DCE spontaneously dissociated during geometry optimization. The higher barriers for configurations with Cl atoms initially oriented toward the S atoms suggest that the proximity of an S atom hinders the cleavage of C–Cl bonds due to steric effects.

### 3.5 Inhibition of PCE and *cis*-DCE Dechlorination Is More Pronounced at a Regularly Sulfidated Fe Surface with Low S Coverage

While the emergence of reaction barriers for PCE chemisorption and *cis*-DCE dechlorination at the Fe sites adjacent to single S atoms may explain why their degradation by S-(n)ZVI proceeds slower compared to TCE and *trans*-DCE,^10,11^ the calculated barriers are too small for a fully quantitative assessment of the CE reactivity trends at these sites. Furthermore, some of the calculated barriers completely disappeared after correction for ZPE (Figure S14). Therefore, we further examined a regularly sulfidated Fe(110) surface, where 1/8 of Fe hollow sites were doped with S atoms (termed as “S_1/8 ML_-Fe(110)”). This surface model, analogous to regularly sulfidated Fe surfaces constructed in our previous study,^29^ has lower S coverage, at which both S and Fe sites are reasonably accessible for CE molecules. Such a model can be considered representative of S-nZVI particles prepared by the postsulfidation method with the S/Fe mole ratio of 0.007, assuming the particle BET specific surface area of 32.4 m^2^ g^–1^ and the deposition of all S atoms in a single atomic layer.^26^ However, experimental data showed that S atoms are distributed within a several nm-thick layer on the surface of postsulfidated S-nZVI,^26^ resulting in the dilution of S coverage. This implies that the S_1/8 ML_-Fe(110) model could also be representative of particles with higher S/Fe ratios.

Geometry optimizations of adsorption complexes at the S_1/8 ML_-Fe(110) surface showed different results for individual CEs (Figure S15). PCE and TCE formed stable physisorption complexes, in which the molecules were adsorbed above a Fe site between the S atoms in a tilted configuration. The C–Fe distances in these complexes were >3 Å. In contrast, DCE isomers chemisorbed directly at the Fe site (C–Fe distances ∼2 Å), with *trans*-DCE undergoing spontaneous cleavage of both C–Cl bonds. These differences are also reflected in the adsorption energies, which reached −180 kJ mol^–1^ for DCE isomers and only −80 kJ mol^–1^ for PCE and TCE (Table S2). These findings are in agreement with the hindered PCE chemisorption at Fe sites near single S atoms, indicating that the steric effects of S atoms influence the S-(n)ZVI surface reactivity with CEs. At the chosen S coverage, the Fe site between S atoms remained freely accessible for DCE isomers, while exhibiting steric hindrance to the larger TCE and PCE molecules.

**Figure 4:**
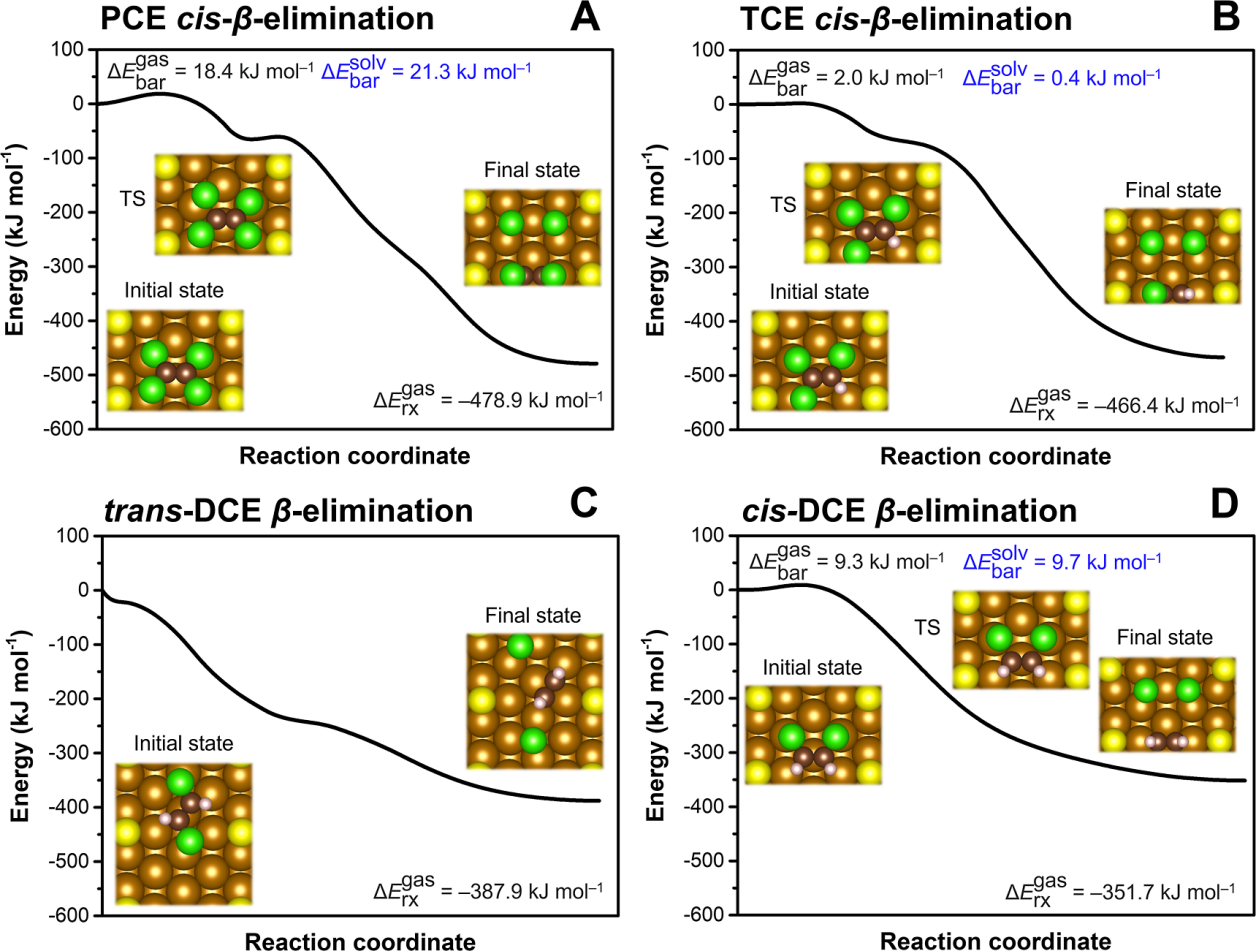
Reaction profiles of chloroethene *β*-dichloroelimination reactions at the S_1/8 ML_-Fe(110) surface: (A) PCE, (B) TCE, (C) *trans*-DCE, and (D) *cis*-DCE. CI-NEB calculations were performed in the gas phase (values in black). The solvent effect on the reaction barrier was included using a continuum solvation model with the structures of reactants and transition states taken from the CI-NEB calculation (values in blue). TS denotes the transition state. The chemisorbed state in panel (C) was calculated with fixed C–Cl distances to prevent spontaneous cleavage of Cl atoms during structural relaxations.

CI-NEB calculations offered additional insights into the extent of S-induced effects on CE dechlorination at this surface. While the *β*-dichloroelimination barrier significantly increased to 18.4 kJ mol^–1^ for PCE, it remained negligible for TCE (Figure 4A,B). This highlights that the steric effects of nearby S atoms hinder the degradation of the bulkier PCE molecules more severely compared to TCE. When the PCE and TCE reaction pathways were divided into separate chemisorption and *β*-elimination steps, an even higher barrier for PCE was observed (Figure S16A), providing additional support for hindered PCE chemisorption at Fe sites near S atoms. The PCE *β*-dichloroelimination barrier was significant even after correcting for ZPE (Figure S17). In contrast, the TCE displayed a smooth transition from the physisorbed to the chemisorbed state with a negligible barrier (Figure S16B). The different extents of steric hindrance for individual CE molecules were also reflected in the displacements of S atoms from their original positions upon CE chemisorption (Table S3), with the average displacement decreasing in the order PCE ≫ TCE > *cis*-DCE > *trans*-DCE.

The *β*-dichloroelimination profile for *trans*-DCE did not reveal any barrier (Figure 4C) when the chemisorbed complex optimized with fixed C–Cl bond lengths was used as the initial state. Among all of the investigated CEs, the geometry of the *trans*-DCE molecule was the most favorable for interaction with the S_1/8 ML_-Fe(110) surface as both its chemisorption and the cleavage of Cl atoms were not constrained by the nearby S atoms.

The *cis*-DCE *β*-dichloroelimination displayed a barrier of ∼10 kJ mol^–1^ (Figure 4D), which is higher than that calculated at the Fe sites adjacent to a single S atom discussed above. This increase in the reaction barrier can be attributed to the combination of steric effects (Table S3) and dilution of the electron density at Fe sites due to electron transfer to S atoms (Figure S18). Based on Bader charge analysis, the loss of electron density at the Fe sites interacting with the C=C bond and Cl atoms of CEs was 0.02 |*e*| compared to the atop Fe sites on the pristine Fe(110) surface. Although a similar decrease in electron density was found at the Fe atom adjacent to the S-on-Fe(110) site,^29^ no decrease was apparent at more distant Fe sites. Higher S coverage thus causes a decrease in electron density at a larger number of Fe sites, which may negatively affect electron transfer to the adsorbed molecules. The degradation of c*is*-DCE might be more severely hindered by this charge redistribution, given that *cis*-DCE has a lower affinity for electron transfer (i.e., higher *E*_LUMO_ value) compared to the other investigated CEs.

The calculated *β*-dichloroelimination barriers at the regularly sulfidated Fe surface with low S coverage, exemplified by the S_1/8 ML_-Fe(110) surface model, provide insights into the observed slower degradation of PCE and *cis*-DCE with S-(n)ZVI in experimental studies.^10−12^ The lack of correlation between the observed reaction rates and the *E*_LUMO_ values of CEs in experiments with S-(n)ZVI may stem from the steric effects of S atoms on nearby reactive Fe sites. As shown in previous sections, CEs undergo dechlorination reactions more easily at Fe sites compared to S sites, provided they can chemisorb to the Fe site. Assuming that Fe sites farther away from S atoms will be oxidized in aqueous environments and thus not be available for efficient electron transfer, the steric effects of S atoms on nearby Fe atoms likely control the selectivity of S-(n)ZVI by regulating the accessibility of Fe sites to contaminants. At higher S coverage, fewer Fe sites will be accessible to any of the CE molecules, making surface reactivity more influenced by the reactivity of S sites, discussed in detail in Sections 3.2 and 3.3. This aligns with the experimentally reported higher relative reactivity of *cis*-DCE with S-(n)ZVI particles of low S/Fe ratio and, in turn, the higher relative reactivity of PCE with particles of high S/Fe ratio.^11,12^ Further increase in S coverage will ultimately lead to suppression of dechlorination reactions, as shown in our recent study with TCE.^29^ These findings fully agree with the experimentally observed existence of an optimal sulfur loading.^5,11,30^

### 3.6 Assumptions and Limitations of This Study

Several assumptions and limitations of the molecular modeling exercise performed here should be acknowledged. First, the use of the straight Fe(110) surface as a model does not encompass all possible structural features present on the surface of S-(n)ZVI, such as vacancies, steps, or kinks. These sites, with lower Fe atom coordination numbers, could exhibit higher reactivity for dechlorination reactions. For instance, recent research has indicated that stepped Fe surfaces play a crucial role in controlling iron corrosion in aqueous media.^62^ However, it is reasonable to expect that sulfidation will preferentially block these highly reactive sites, considering earlier evidence showing the deactivation of step edges by S atoms on Ni surfaces.^63^ The reactivity of such S sites is expected to exhibit trends similar to those described in the present study for the two S sites with different architectures.

Second, the surfaces of the (n)ZVI and S-(n)ZVI particles in real remediation scenarios are unlikely to remain pristine. Upon contact with water, (n)ZVI rapidly develops a surface passivation layer formed by iron (oxyhydr)oxides.^64,65^ Nonetheless, prior research has shown that sulfidation effectively hinders (n)ZVI surface corrosion by preventing the adsorption of water and hydrogen atoms at the S sites and nearby Fe sites.^5,6,28,29,49^ Based on these findings, the S_1/8 ML_-Fe(110) model used in this study could potentially represent a convenient reaction site for CE dechlorination at the surface of postsulfidated S-(n)ZVI. In this model, the chosen S coverage is expected to decrease the interactions between the surface and water molecules (i.e., hydrophilicity) while maintaining the accessibility of Fe sites for electron transfer toward contaminants. Considering that oxidation decreases (n)ZVI reactivity,^29,64,66^ it can be anticipated that the particle reactivity will be to a large degree controlled by the abundance of reactive (unoxidized) Fe sites, exemplified by the S_1/8 ML_-Fe(110) model. To fully understand the effects of surface corrosion on the reactivity and selectivity of (n)ZVI materials toward CEs, mechanistic studies exploring dechlorination reactions at Fe surfaces with different extents of oxidation are needed.

Third, this study does not explicitly simulate water molecules due to the complexity and computational demands of such calculations. Instead, we adopted an implicit solvent approach, representing solvent molecules as polarizable continuous medium with a specific electric conductivity. This approximation may not account for potential specific solvent-adsorbate and solvent–surface interactions such as hydrogen bonds. Nevertheless, we expect the impact of this approximation to be minor, given the relatively hydrophobic nature of S sites, including nearby Fe atoms and CE molecules.

Lastly, this study deals only with dechlorination reactions controlled by direct electron transfer (i.e., *β*-elimination). Recently, H*_ads_-mediated hydrogenolysis was suggested to play a significant role in S-(n)ZVI systems, especially in the degradation of CEs with a lower degree of chlorination at low S surface coverage.^10,12^ While this study attempts to explain the observed CE reactivity trends without accounting for hydrogenolysis, the H*_ads_-mediated reactions could potentially contribute to controlling the S-(n)ZVI reactivity. However, the mechanism by which sulfidation would promote H*_ads_ generation or retention at the S-(n)ZVI surface remains unclear. Contradictory effects of S atoms on the stability of H*_ads_ have been reported. Several studies showed that S atoms hinder H* adsorption at the Fe surface^5,29,49^ and that H*-mediated reactions, such as acetylene hydrogenation and chloramphenicol denitration, were inhibited by (n)ZVI sulfidation.^4,23,24,26,30,67^ In contrast, a higher H*_ads_ abundance at the surface of S-nZVI compared to pristine nZVI has been observed in another study.^68^ To gain a comprehensive understanding of all processes governing the selectivity of S-(n)ZVI for individual CEs, more mechanistic insights are needed into the formation, stability, and recombination of H*_ads_ at S-(n)ZVI surfaces with varying degrees of sulfidation, along with their reactivity with various CEs.

## 4 Conclusions

This study explores the reductive dechlorination pathways of CEs on the S-(n)ZVI surface at the atomic scale. The electron-transfer-controlled *β*-dichloroelimination reactions were found to be both kinetically and thermodynamically more favorable at Fe sites compared to S sites. At Fe sites adjacent to S atoms, which are less likely to be oxidized in aqueous environments and thus are accessible to contaminants, the selectivity of S-(n)ZVI for CE molecules is controlled by the interplay between the affinity of individual CEs for electron transfer and the steric hindrance imposed by S atoms. Consequently, TCE and *trans*-DCE undergo faster dechlorination at these sites than PCE and *cis*-DCE. At the S site, dechlorination barriers are governed by the affinity of CEs for electron transfer (i.e., correlate with *E*_LUMO_ values). Comparison of the CE reactivity patterns at the flat S-in-Fe(110) and elevated S-on-Fe(110) sites further reveals that the architecture of the reactive site can alter the preferential stereochemistry of the *β*-dichloroelimination reaction (*cis* vs *trans*).

The interplay of the varying reactivities of Fe and S sites with individual CEs provides an explanation for the observed selectivity of S-(n)ZVI materials for individual CEs, without considering H*_ads_-mediated reactions. The significance of indirect reduction pathways in CE dechlorination deserves further investigation. A detailed understanding of the structure–reactivity relationships governing the performance of S-(n)ZVI in contaminant removal can streamline the tailored design of highly efficient materials for groundwater cleanup.

## Data Availability

The data from DFT calculations underlying this study are openly available in Zenodo at https://zenodo.org/records/10663010.

## Supporting Information Available

Computational details; configurations of adsorbed CEs with geometric parameters; energy profile of PCE chemisorption at the Fe(110) surface; *β*-dichloroelimination profiles of CE dechlorination at the Fe(110) surface and Fe sites of S-in-Fe(110) and S-on-Fe(110) surface models; chemisorption and *β*-dichloroelimination profiles of PCE and TCE at the regularly sulfidated S_1/8 ML_-Fe(110) surface; CE adsorption energies in the gas phase and solvent with contributions from pure DFT (PBE) and D3 dispersion correction; ZPE-corrected reaction barriers and their correlation with *E*_LUMO_; comparisons between PBE+D3 and SCAN-calculated reaction barriers; projected density of states plots of the adsorbed CE molecules at the S-in-Fe(110) site and the adjacent Fe site; displacement of S atoms in the S_1/8 ML_-Fe(110) surface model upon chemisorption of CE molecules; and charge density redistribution on the Fe(110) surface induced by S adatoms (PDF)
