# Supplementary material for: Reductive Dechlorination of Chlorinated Ethenes at the Sulfidated Zero-Valent Iron Surface: A Mechanistic DFT Study
Source: J Phys Chem C Nanomater Interfaces. 2024 Feb 28;128(10):4180–91. doi: 10.1021/acs.jpcc.4c00865 (PMC10945477; doi:10.1021/acs.jpcc.4c00865)
Supplement: Supplementary file 1 — jp4c00865_si_001.pdf [file jp4c00865_si_001.pdf]

Supporting Information

# Reductive Dechlorination of Chlorinated Ethenes at the Sulfidated Zero-Valent Iron Surface: A Mechanistic DFT Study

*Miroslav Brumovský* [a, *] *and Daniel Tunega* [a]

[a] University of Natural Resources and Life Sciences, Vienna, Department of Forest- and Soil Sciences, Institute of Soil Research, Peter-Jordan-Straße 82, 1190 Vienna, Austria

* Corresponding author

E-mail address: miroslav.brumovsky@boku.ac.at

<u>Summary</u>

Number of pages: 23

Number of tables: 3

Number of figures: 18

## Text S1. Computational details

### Calculation of adsorption energies and reaction barriers

The adsorption energy ($\Delta E_{ads}$) of CEs at modeled surfaces was calculated as

$$\Delta E_{ads} = E_{complex} - (E_{surf} + E_{mol}), \quad (1)$$

where $E_{complex}$ is the total electronic energy of the CE molecule adsorbed at the surface, $E_{slab}$ is the total electronic energy of the bare surface, and $E_{mol}$ is the total electronic energy of the isolated CE molecule in the vacuum. A negative $E_{ads}$ corresponds to a stable CE-surface complex.

The energy profiles of CE dechlorination reactions were calculated for the $\beta$-dichloroelimination pathways using the climbing image nudged elastic band (CI-NEB) method.[1] The reaction coordinates were partitioned into nine images and relaxed until the change in the energy barrier was smaller than 0.005 eV. The calculation was repeated with two additional images when no barrier appeared. The energy barrier ($\Delta E_{bar}$) was calculated as the difference between the total electronic energy of the transition state geometry ($E_{TS}$) and the reactant geometry ($E_{react}$) as

$$\Delta E_{bar} = E_{TS} - E_{react}. \quad (2)$$

We found in our previous study that the interactions of CEs with the S sites of sulfidated (n)ZVI surfaces can be mainly attributed to weak dispersion forces,[2] which were treated in our calculations using the empirical D3 correction with Becke-Johnson damping.[3,4] To verify the accuracy of the PBE+D3 approach, the energy barriers were also calculated with the accurate meta-GGA strongly constrained and appropriately normed (SCAN) functional,[5] which inherently accounts for short- and medium-range dispersion forces. This benchmarking was performed using single-point calculations on the PBE+D3-optimized geometries of reactants and transition states.

The reaction energy ($\Delta E_{rx}$) was calculated as the difference between the total electronic energies of the product ($E_{prod}$) and reactant ($E_{react}$) geometries as

$$\Delta E_{rx} = E_{prod} - E_{react}. \quad (3)$$

The optimized structures of adsorption complexes and transition states were visualized using VESTA 3.[6]

### Zero-point energy correction

The energy barrier ($\Delta E_{bar}$) was corrected for the zero-point energy (ZPE) vibrations of reactants and transition states as

$$\mathrm{ZPE} = \sum_i^{\alpha} \frac{1}{2} h\nu_i, \quad (4)$$

where $v_i$ is the vibrational frequency of the $i^{th}$ normal mode in Hz and $\alpha$ is the number of vibrational degrees of freedom. Imaginary frequencies detected in transition states were not included in the ZPE calculation. To ensure high accuracy of frequency analyses, we compared the calculated vibrational frequencies for gas-phase TCE with experimental data from ref. 7, reaching r = 0.99978.

**Charge analysis and calculations of density of states**

Bader charge analysis was conducted using the program Bader[8,9] implemented into VASP to understand the electron transfer between S atoms and the Fe surface. Density of states (DOS) calculations were done using the tetrahedron smearing method with Blöchl corrections.[10] The post-processing of DOS calculations was performed using the program VASPKIT.[11]

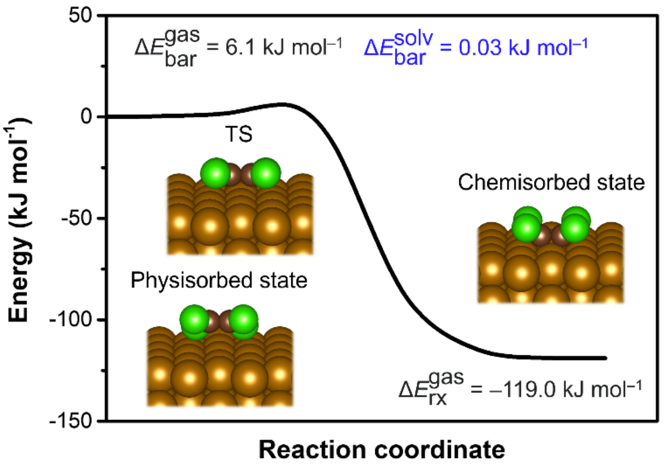


**Figure S1.** Reaction profile of PCE chemisorption at the pristine Fe(110) surface. CI-NEB calculations were performed in the gas phase (values in black). The solvent effect on the reaction barrier was included using a continuum solvation model with the structures of reactants and transition states taken from the CI-NEB calculation (value in blue). The data were taken from ref. 12.

*Comments to Figure S1*

In the physisorbed state, the PCE molecule was titled with respect to the Fe(110) surface. The geometry of PCE in the physisorption complex showed only minor changes in geometry compared to the gas phase, with the C-Cl bonds oriented toward the Fe slab elongated by ~0.03 Å (Table S1). The calculated reaction barrier for the transition from the physisorbed to the chemisorbed complex was only of 6.1 kJ mol$^{-1}$ in the gas phase and it virtually disappeared when solvent effects were included in the calculation.[12]

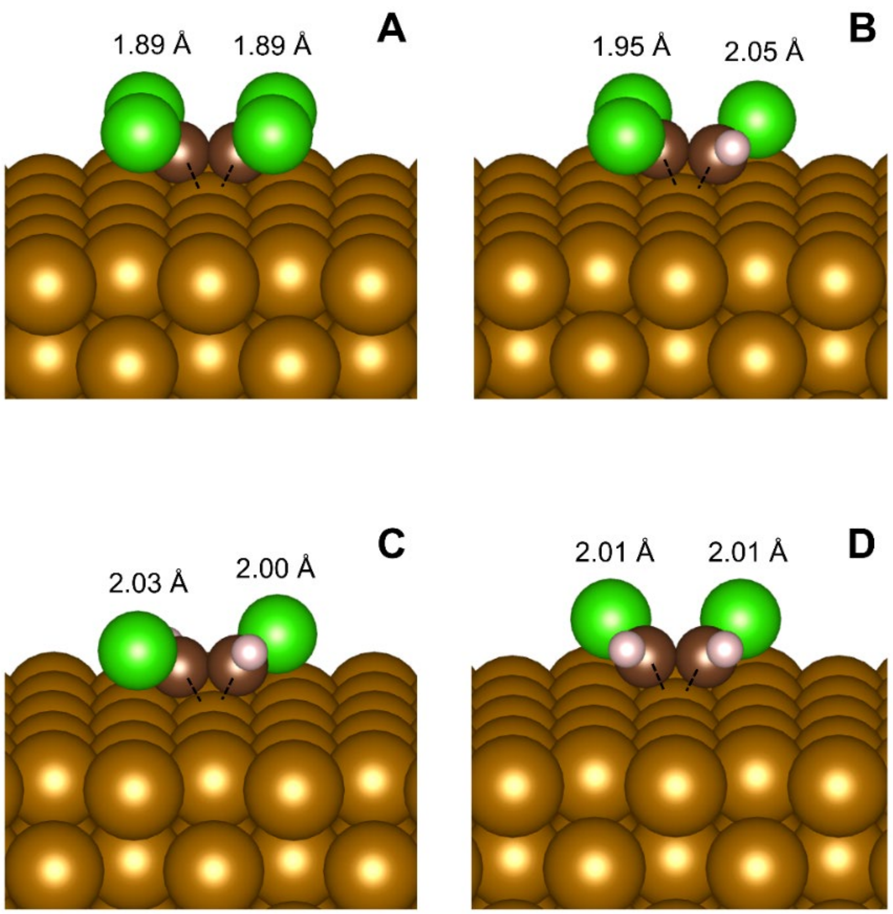


**Figure S2.** PBE+D3-optimized configurations of chlorinated ethenes adsorbed at the pristine Fe(110) surface with shown C–Fe distances: (A) chemisorbed PCE, (B) TCE, (C) *trans*-DCE, and (D) *cis*-DCE. The adsorption complexes in (B–D) were optimized with frozen C–Cl distances to prevent spontaneous Cl dissociation. Configurations of PCE and TCE in (A) and (B) were adapted from refs. 2 and 12.

**Table S1.** Geometry of chloroethene molecules in the gas phase and adsorbed at the modeled surfaces and sites.

| Structure/adsorption complex | Bond length (Å) | | | | | Dihedral angle* (°) | |
|---|---|---|---|---|---|---|---|
| | C=C | C–Cl1† | C–Cl2† | C–Cl3† | C–H | Cl–C–C–Cl | H–C–C–X |
| *Gas phase* | | | | | | | |
| PCE‡ | 1.355 | 1.710 | -- | -- | -- | 179.9 | -- |
| TCE§ | 1.343 | 1.721 | 1.708 | 1.707 | 1.089 | 180.0 | 180.0 |
| *trans*-DCE | 1.335 | 1.720 | -- | -- | 1.090 | 180.0 | 180.0 |
| *cis*-DCE‡ | 1.337 | 1.710 | -- | -- | 1.090 | -- | 180.0 |
| *Pristine Fe(110)* | | | | | | | |
| PCE (physisorbed) ‡ | 1.359 | 1.698, 1.743¶ | -- | -- | -- | 184.7, 185.2 | -- |
| PCE (chemisorbed) ‡ | 1.447 | 1.852–1.879 | -- | -- | -- | 125.5, 126.8 | -- |
| TCE§ | 1.485 | 1.721 (fixed) | 1.708 (fixed) | 1.707 (fixed) | 1.113 | 130.8 | 133.0 |
| *trans*-DCE | 1.470 | 1.720 (fixed) | -- | -- | 1.098, 1.107 | 134.4 | 126.8 |
| *cis*-DCE | 1.485 | 1.710 (fixed) | -- | -- | 1.110, 1.111 | -- | 131.3, 131.2 |
| *S-in-Fe(110) site (S site)* | | | | | | | |
| PCE | 1.361 | 1.719 | -- | -- | -- | 195.2 | -- |
| TCE§ | 1.345 | 1.706 | 1.733 | 1.723 | 1.089 | 187.8 | 185.5 |
| *trans*-DCE | 1.337 | 1.730 | -- | -- | 1.091 | 193.9 | 183.4 |
| *cis*-DCE | 1.336 | 1.727 | -- | -- | 1.090 | -- | 184.1 |
| *S-on-Fe(110) site (S site)* | | | | | | | |
| PCE | 1.353 | 1.696, 1.751¶ | -- | -- | -- | 183.9 | -- |
| TCE§ | 1.343 | 1.704 | 1.743 | 1.713 | 1.089 | 183.9 | 183.2 |
| *trans*-DCE | 1.334 | 1.712, 1.746¶ | -- | -- | 1.088, 1.090¶ | 181.8 | 182.4 |
| *cis*-DCE | 1.335 | 1.726 | -- | -- | 1.089 | -- | 182.9 |

**Table S1 – cont.**

| Adsorption complex | Bond length (Å) | | | | | Dihedral angle* (°) | |
|---|---|---|---|---|---|---|---|
| | C=C | C–Cl1† | C–Cl2† | C–Cl3† | C–H | Cl–C–C–Cl | H–C–C–X |
| *Fe site adjacent to S-in-Fe(110)* | | | | | | | |
| PCE (physisorbed) | 1.364 | 1.708–1.723 | -- | -- | -- | 185.9 | -- |
| PCE (chemisorbed) | 1.489 | 1.710 (fixed) | -- | -- | -- | 134.7, 135.5 | -- |
| TCE§ | 1.485 | 1.721 (fixed) | 1.708 (fixed) | 1.707 (fixed) | 1.112 | 131.9 | 133.2 |
| *trans*-DCE | 1.469 | 1.720 (fixed) | -- | -- | 1.102 | 133.6 | 128.6 |
| *cis*-DCE (Cl twds. Fe) | 1.472 | 1.851 | -- | -- | 1.110 | -- | 124.8, 124.9 |
| *cis*-DCE (Cl twds. S) | 1.482 | 1.847 | -- | -- | 1.108 | -- | 125.7 |
| *Fe site adjacent to S-on-Fe(110)* | | | | | | | |
| PCE (physisorbed) | 1.358 | 1.697, 1.744¶ | -- | -- | -- | 184.3 | -- |
| PCE (chemisorbed) | 1.487 | 1.710 (fixed) | -- | -- | -- | 134.0, 134.2 | -- |
| TCE§ | 1.487 | 1.721 (fixed) | 1.708 (fixed) | 1.707 (fixed) | 1.126 | 133.8 | 127.4 |
| *trans*-DCE | 1.471 | 1.720 (fixed) | -- | -- | 1.096, 1.113 | 133.7 | 127.6 |
| *cis*-DCE (Cl twds. Fe) | 1.476 | 1.862 | -- | -- | 1.106 | -- | 124.6, 124.8 |
| *cis*-DCE (Cl twds. S) | 1.481 | 1.846 | -- | -- | 1.112 | -- | 125.3, 125.4 |
| $S_{1/8\ ML}$*-Fe(110)* | | | | | | | |
| PCE (physisorbed) | 1.356 | 1.697–1.734¶ | -- | -- | -- | 181.1, 181.5 | -- |
| PCE (chemisorbed) | 1.457 | 1.819–1.849 | -- | -- | -- | 122.9, 126.3 | -- |
| TCE (physisorbed) | 1.349 | 1.708 | 1.712 | 1.723 | 1.089 | 180.2 | 181.4 |
| TCE (chemisorbed) | 1.476 | 1.721 (fixed) | 1.708 (fixed) | 1.707 (fixed) | 1.108 | 129.5 | 131.4 |
| *trans*-DCE | 1.474 | 1.720 (fixed) | -- | -- | 1.098, 1.101 | 142.4 | 119.4 |
| *cis*-DCE | 1.468 | 1.833 | -- | -- | 1.104 | -- | 125.9, 126.2 |

* Values <180° indicate the concave orientation of atoms relative to the surface, while values >180° indicate convex orientation.

† The lengths of C-Cl bonds in the symmetric molecules of PCE, *trans*-DCE, and *cis*-DCE are shown in the C-Cl1 column. Atomic positions for TCE are as follows:

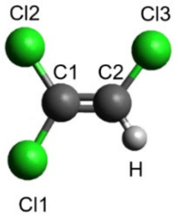


‡ Values taken from ref. 12.

§ Values taken from ref. 2.

¶ The C-Cl and C-H bonds oriented toward the slab were longer than the C-Cl and C-H bonds oriented toward vacuum/solvent.

**Table S2.** PBE+D3-calculated adsorption energies of chlorinated ethenes at various surfaces and sites in the gas phase ($\Delta E_{\mathrm{ads}}^{\mathrm{gas}}$) and in the implicit solvent ($\Delta E_{\mathrm{ads}}^{\mathrm{solv}}$) in kJ mol$^{-1}$ with shown contributions of pure DFT ($\Delta E_{\mathrm{ads}}^{\mathrm{DFT}}$) and empirical dispersion correction ($\Delta E_{\mathrm{ads}}^{\mathrm{disp}}$) to the total adsorption energy in the gas phase ($\Delta E_{\mathrm{ads}}^{\mathrm{gas}} = \Delta E_{\mathrm{ads}}^{\mathrm{DFT}} + \Delta E_{\mathrm{ads}}^{\mathrm{disp}}$). The adsorption complexes labeled with * were optimized with frozen C–Cl distances to prevent spontaneous Cl dissociation.

| **Adsorption complex** | $\boldsymbol{\Delta E_{\mathrm{ads}}^{\mathrm{gas}}}$ | $\boldsymbol{\Delta E_{\mathrm{ads}}^{\mathrm{DFT}}}$ | $\boldsymbol{\Delta E_{\mathrm{ads}}^{\mathrm{disp}}}$ | $\boldsymbol{\Delta E_{\mathrm{ads}}^{\mathrm{solv}}}$ |
|---|---|---|---|---|
| Fe(110)···PCE (phys) † | –81.1 | 6.6 | –87.8 | –92.6 |
| Fe(110)···PCE (chem) † | –200.1 | –88.6 | –111.5 | –206.3 |
| Fe(110)···TCE*, ‡ | –165.6 | –68.8 | –96.8 | –174.8 |
| Fe(110)···tDCE* | –169.8 | –89.0 | –80.8 | –177.8 |
| Fe(110)···cDCE* | –165.9 | –84.2 | –81.6 | –171.6 |
| S-in-Fe(110): S site···PCE | –89.9 | 12.6 | –102.5 | –102.1 |
| S-in-Fe(110): S site···TCE‡ | –81.8 | –3.4 | –78.3 | –89.7 |
| S-in-Fe(110): S site···tDCE | –65.6 | 3.2 | –68.8 | –71.3 |
| S-in-Fe(110): S site···cDCE | –75.2 | –9.6 | –65.6 | –84.1 |
| S-in-Fe(110): Fe site···PCE (phys) | –80.6 | 14.7 | –95.3 | –92.4 |
| S-in-Fe(110): Fe site···PCE (chem)* | –157.4 | –46.0 | –111.5 | –167.6 |
| S-in-Fe(110): Fe site···TCE*, ‡ | –166.1 | –68.4 | –97.6 | –173.2 |
| S-in-Fe(110): Fe site···tDCE* | –168.5 | –87.5 | –81.1 | –175.3 |
| S-in-Fe(110): Fe site···cDCE (Cl twds. Fe) | –189.0 | –112.3 | –76.6 | –189.7 |
| S-in-Fe(110): Fe site···cDCE (Cl twds. S) | –185.0 | –101.8 | –83.2 | –186.3 |
| S-on-Fe(110): S site···PCE | –69.1 | 7.3 | –76.4 | –73.0 |
| S-on-Fe(110): S site···TCE‡ | –62.8 | 3.2 | –66.0 | –67.6 |
| S-on-Fe(110): S site···tDCE | –47.8 | –2.7 | –45.0 | –51.5 |
| S-on-Fe(110): S site···cDCE | –58.2 | 1.7 | –59.9 | –64.8 |
| S-on-Fe(110): Fe site···PCE (phys) | –84.1 | 7.4 | –91.5 | –93.1 |
| S-on-Fe(110): Fe site···PCE (chem)* | –145.0 | –29.1 | –115.8 | –155.0 |
| S-on-Fe(110): Fe site···TCE*, ‡ | –158.8 | –58.4 | –100.4 | –164.1 |
| S-on-Fe(110): Fe site···tDCE* | –167.4 | –83.4 | –83.9 | –172.0 |
| S-on-Fe(110): Fe site···cDCE (Cl twds. Fe) | –182.7 | –97.3 | –85.3 | –182.7 |
| S-on-Fe(110): Fe site···cDCE (Cl twds. S) | –175.6 | –89.4 | –86.1 | –176.1 |
| $S_{1/8\ \mathrm{ML}}$-Fe(110) ···PCE (phys) | –77.5 | 9.1 | –86.6 | –81.2 |
| $S_{1/8\ \mathrm{ML}}$-Fe(110) ···PCE (chem) | –151.3 | –26.3 | –125.0 | –153.4 |
| $S_{1/8\ \mathrm{ML}}$-Fe(110) ···TCE (phys) | –79.4 | 4.9 | –84.2 | –82.4 |
| $S_{1/8\ \mathrm{ML}}$-Fe(110) ···TCE (chem)* | –154.0 | –44.7 | –109.3 | –157.1 |
| $S_{1/8\ \mathrm{ML}}$-Fe(110) ··· tDCE* | –173.4 | –82.6 | –90.9 | –178.6 |
| $S_{1/8\ \mathrm{ML}}$-Fe(110) ··· cDCE | –177.2 | –84.3 | –92.8 | –177.8 |

† Energy values from ref. 12.

‡ Gas phase energy values from ref. 2.

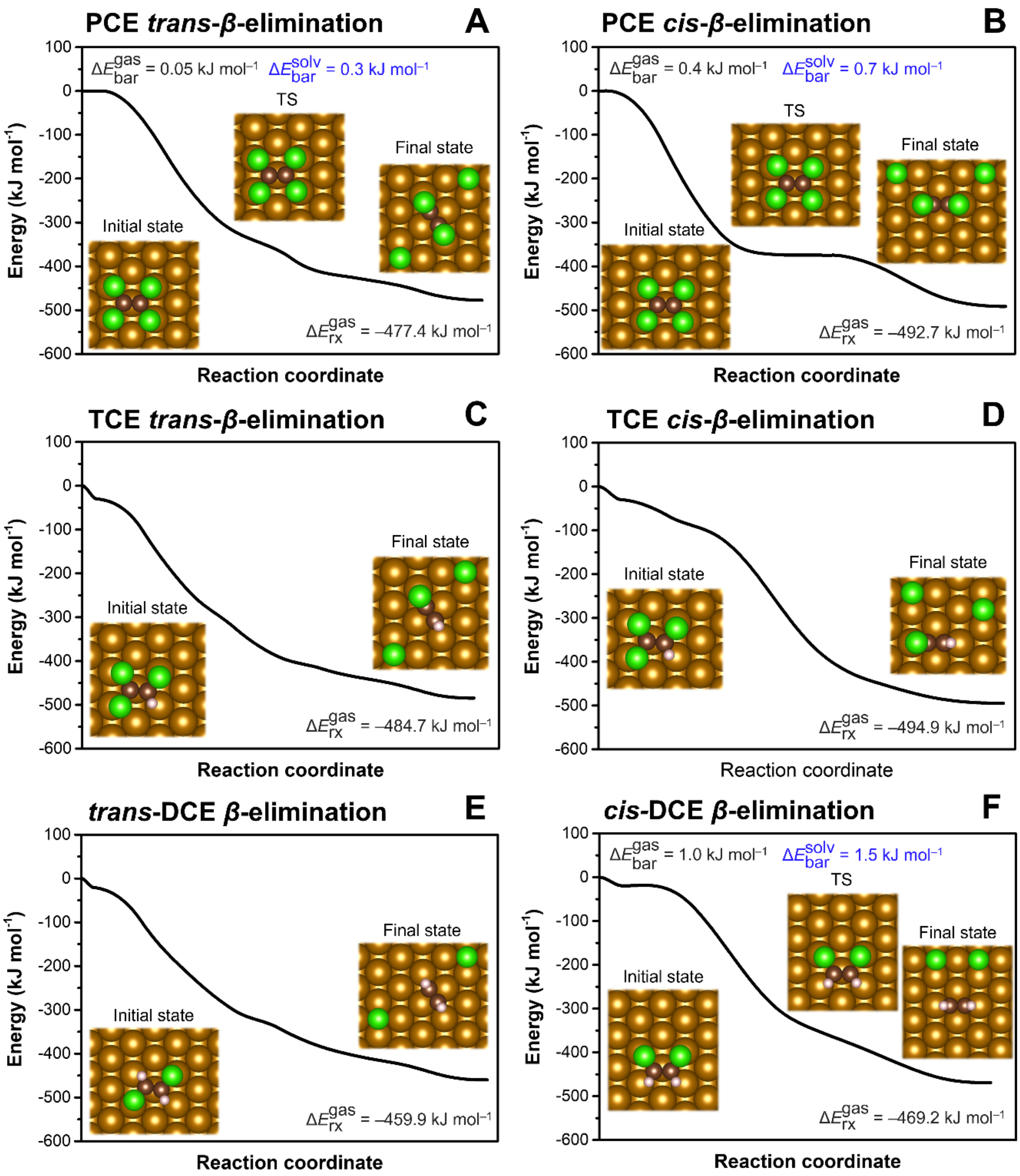


**Figure S3.** Reaction profiles of chloroethene *β*-dichloroelimination reactions at the pristine Fe(110) surface: (A, B) PCE, (C, D) TCE, (E) *trans*-DCE, and (F) *cis*-DCE. CI-NEB calculations were performed in the gas phase (values in black). The initial states in (C–F) were calculated with fixed C-Cl distances to prevent spontaneous cleavage of Cl atoms during structural relaxations. The solvent effect on the reaction barrier was included using a continuum solvation model with the structures of reactants and transition states taken from the CI-NEB calculation (values in blue).

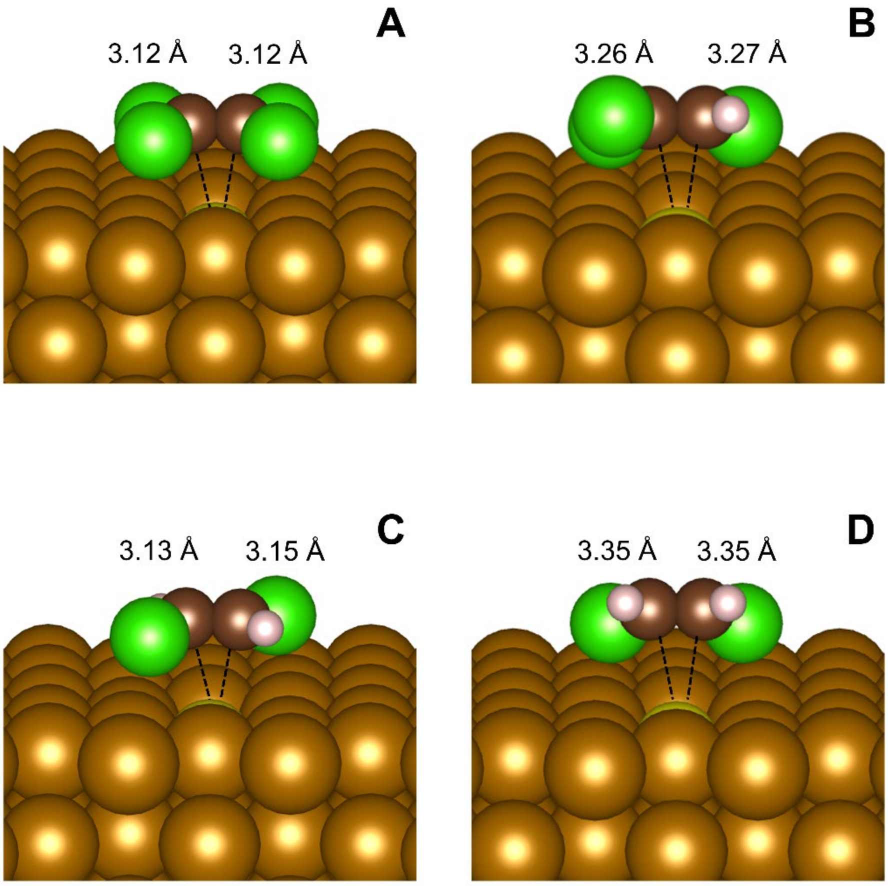


**Figure S4.** PBE+D3-optimized configurations of chlorinated ethenes adsorbed at the S-in-Fe(110) site with shown C–S distances: (A) PCE, (B) TCE, (C) *trans*-DCE, and (D) *cis*-DCE. Configuration of TCE in (B) was adapted from ref. 2.

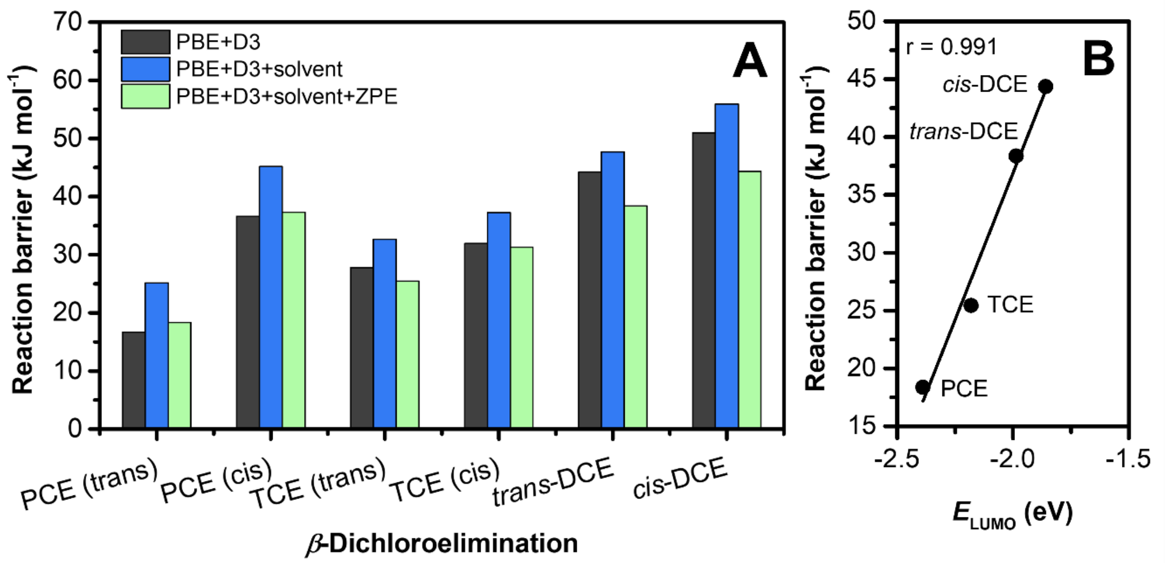


**Figure S5.** *β*-dichloroelimination barriers of chlorinated ethenes at the S-in-Fe(110) site: (A) reaction barriers corrected for implicit solvation and zero-point energy, (B) correlation between $E_{\mathrm{LUMO}}$ of chloroethenes and the barriers for *β*-dichloroelimination reactions. The $E_{\mathrm{LUMO}}$ values were taken from ref. 13.

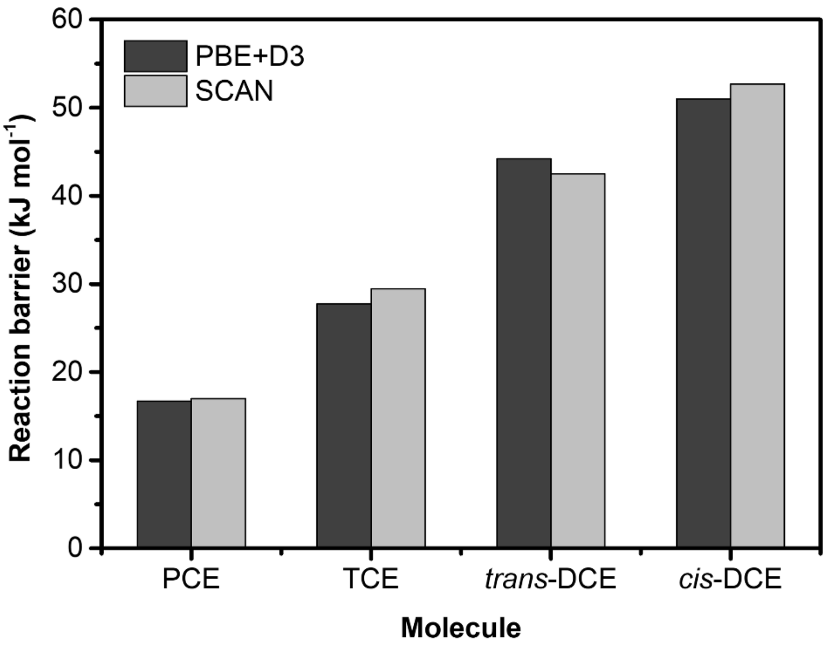


**Figure S6.** Benchmark calculations (PBE+D3 vs. SCAN) of the most favorable *β*-elimination barriers for various chlorinated ethenes at the S-in-Fe(110) site. SCAN energies were obtained from single-point calculations using the PBE+D3 optimized structures of reactants and transition states. Solvent effects and ZPE correction are not included in this comparison.

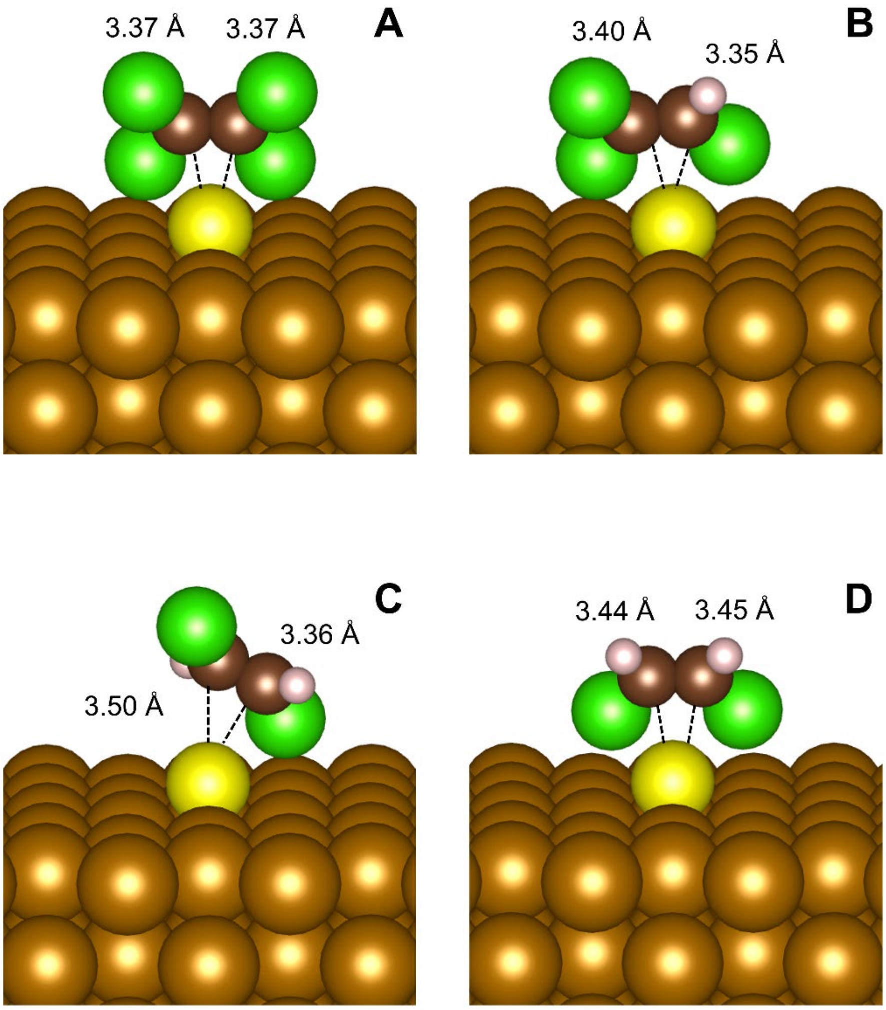


**Figure S7.** PBE+D3-optimized configurations of chlorinated ethenes adsorbed at the S-on-Fe(110) site with shown C–S distances: (A) PCE, (B) TCE, (C) *trans*-DCE, and (D) *cis*-DCE. Configuration of TCE in (B) was adapted from ref. 2.

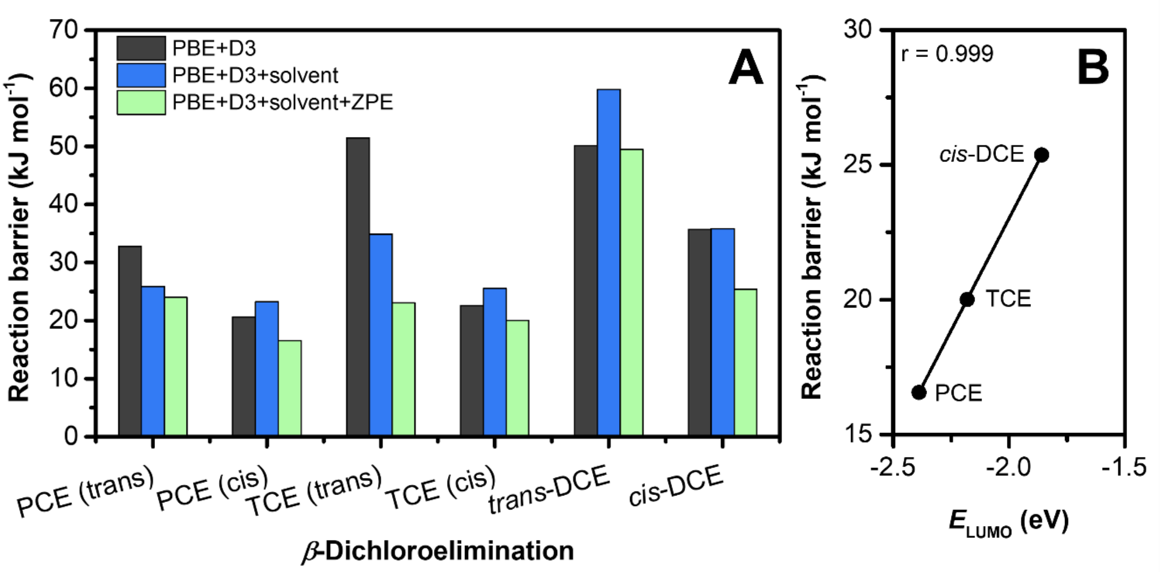


**Figure S8.** *β*-dichloroelimination barriers of chlorinated ethenes at the S-on-Fe(110) site: (A) reaction barriers corrected for implicit solvation and zero-point energy, (B) correlation between $E_{\mathrm{LUMO}}$ of chloroethenes and the barriers for *cis*-*β*-dichloroelimination reactions. The $E_{\mathrm{LUMO}}$ values were taken from ref. 13.

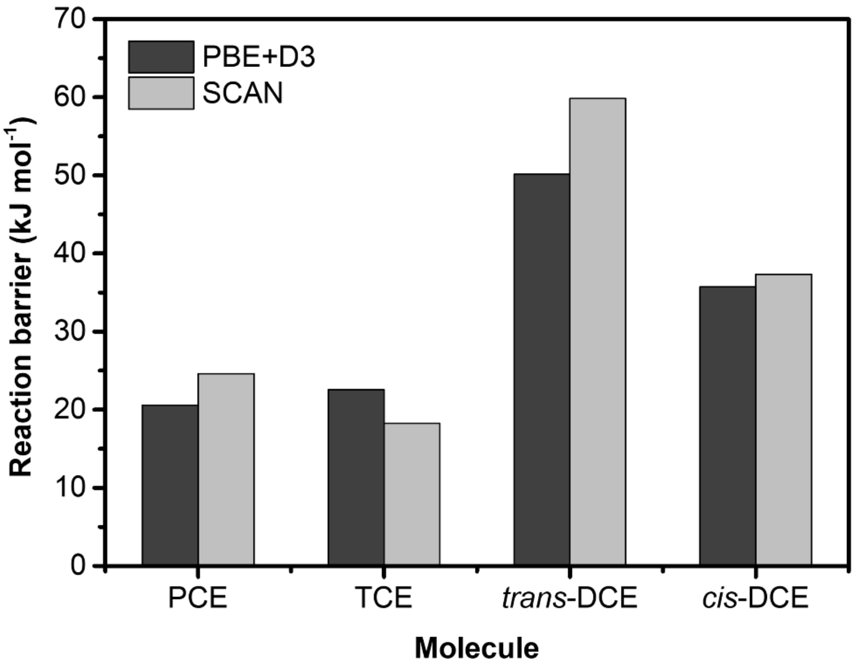


**Figure S9.** Benchmark calculations (PBE+D3 vs. SCAN) of the most favorable *β*-elimination barriers for various chlorinated ethenes at the S-on-Fe(110) site. SCAN energies were obtained from single-point calculations using the PBE+D3 optimized structures of reactants and transition states. Solvent effects and ZPE correction are not included in this comparison.

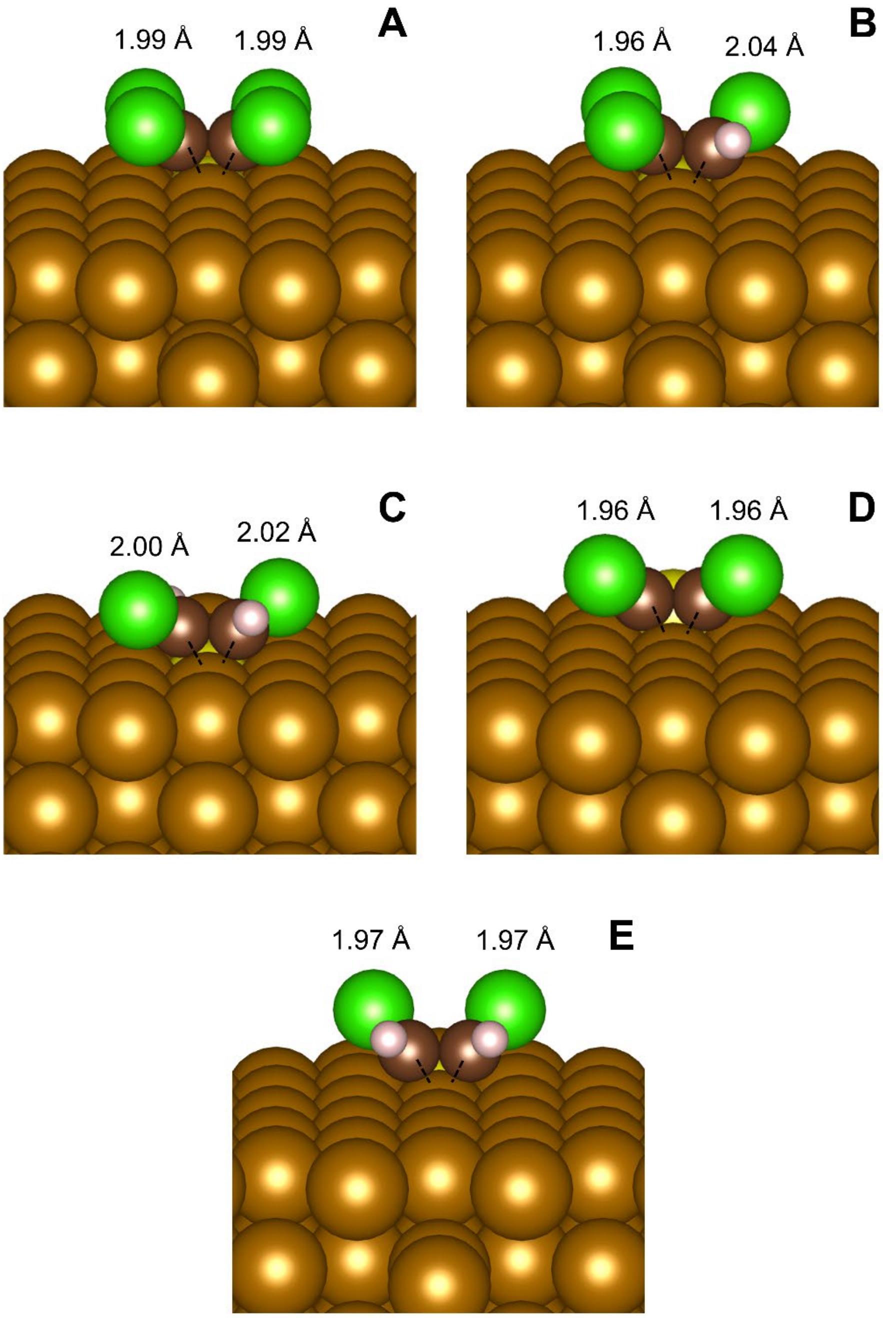


**Figure S10.** DFT+D3-optimized configurations of chlorinated ethenes adsorbed at the Fe site adjacent to the S-in-Fe(110) site with shown C–Fe distances: (A) chemisorbed PCE, (B) TCE, (C) *trans*-DCE, (D) *cis*-DCE with Cl atoms oriented towards the Fe slab, and (E) *cis*-DCE with Cl atoms oriented towards the S atom. The adsorption complexes in (A–C) were optimized with frozen C–Cl distances to prevent spontaneous Cl dissociation. TCE configuration in (B) was adapted from ref. 2.

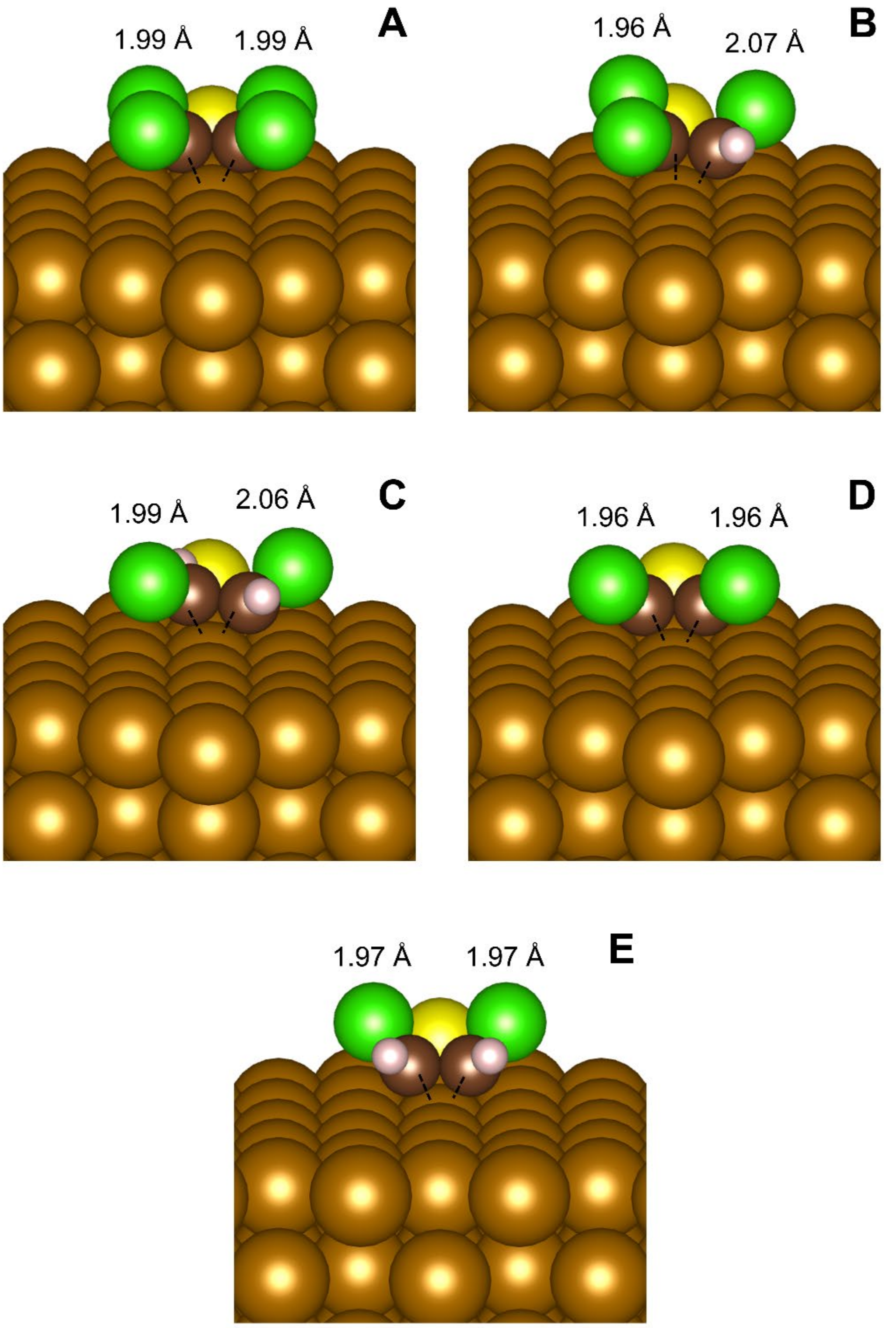


**Figure S11.** DFT+D3-optimized configurations of chlorinated ethenes adsorbed at the Fe site adjacent to the S-on-Fe(110) site with shown C–Fe distances: (A) chemisorbed PCE, (B) TCE, (C) *trans*-DCE, and (D) *cis*-DCE with Cl atoms oriented towards the Fe slab, and (E) *cis*-DCE with Cl atoms oriented towards the S atom. The adsorption complexes in (A–C) were optimized with frozen C–Cl distances to prevent spontaneous Cl dissociation. TCE configuration in (B) was adapted from ref. 2.

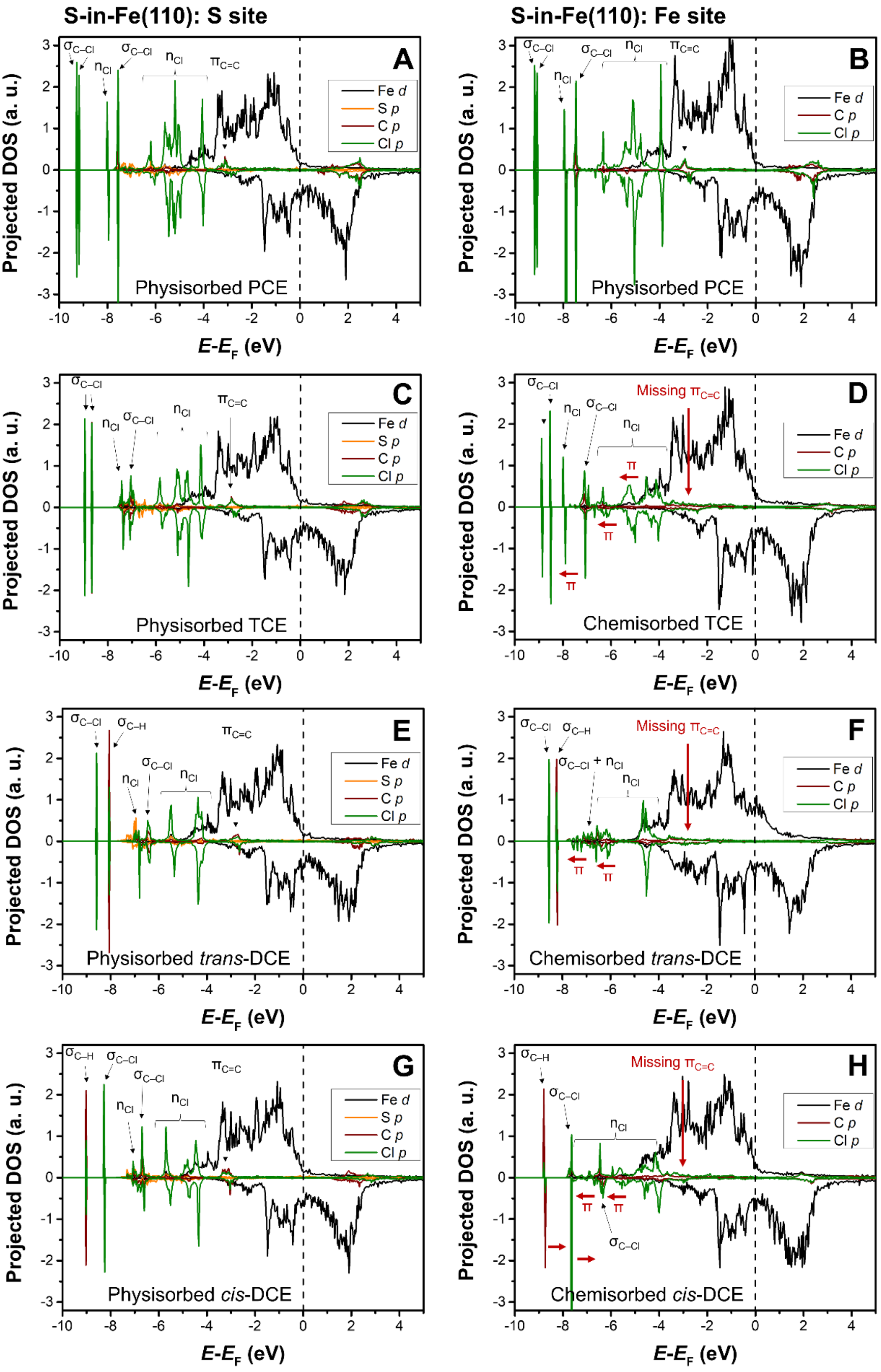


**Figure S12.** Projected DOS of the adsorbed CE molecules at the S-in-Fe(110) site and the adjacent Fe site with the main differences indicated in red: (A, B) PCE, (C, D) TCE, (E, F) *trans*-DCE, and (G, H) *cis*-DCE adsorbed with Cl atoms toward the Fe slab. The geometries in (D) and (F) were calculated with fixed C–Cl distances to prevent spontaneous cleavage of Cl atoms. The assignment of CE

molecular orbitals is based on Khvostenko[14] and verified through the projected DOS for individual atoms and orbitals. The positions of the $n_{Cl}$ and $\sigma_{C\text{–}Cl}$ molecular orbitals in the region –6 to –7 eV of *cis*-DCE and *trans*-DCE are inversed with respect to the gas phase, which is due to a strong interaction of the $n_{Cl}$ orbitals with the slab, resulting in its shift to lower energy. The insets (C) and (D) were adapted from ref. 2.

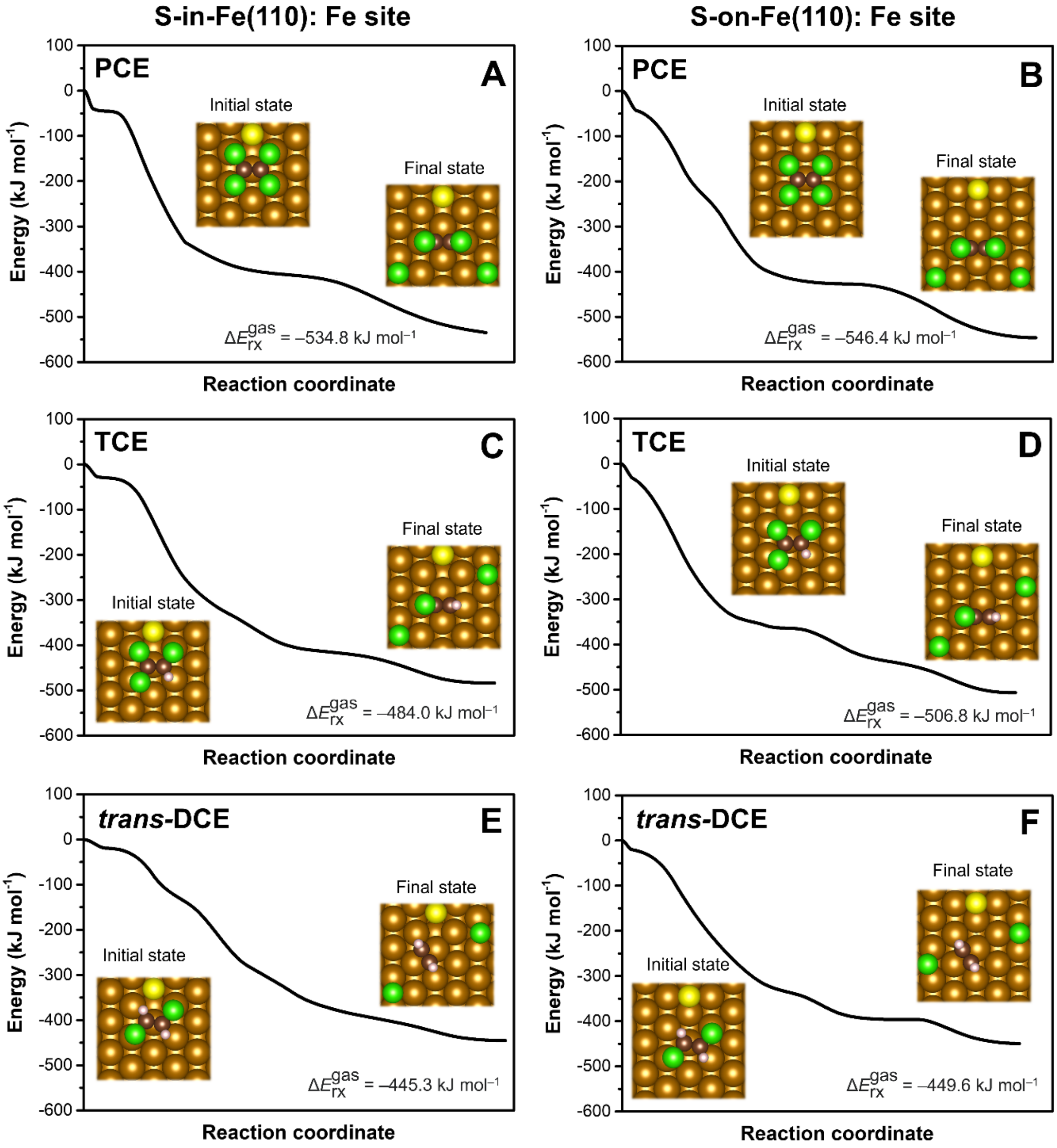


**Figure S13.** Spontaneous dechlorination of various chlorinated ethenes at the Fe sites adjacent to S-in-Fe(110) and S-on(Fe110) sites: (A, B) chemisorbed PCE, (C, D) TCE, and (E, F) *trans*-DCE. CI-NEB calculations were performed in the gas phase. The initial states were calculated with fixed C–Cl distances to prevent spontaneous cleavage of Cl atoms during structural relaxations. TCE dechlorination pathways in panels (C) and (D) were adapted from ref. 2.

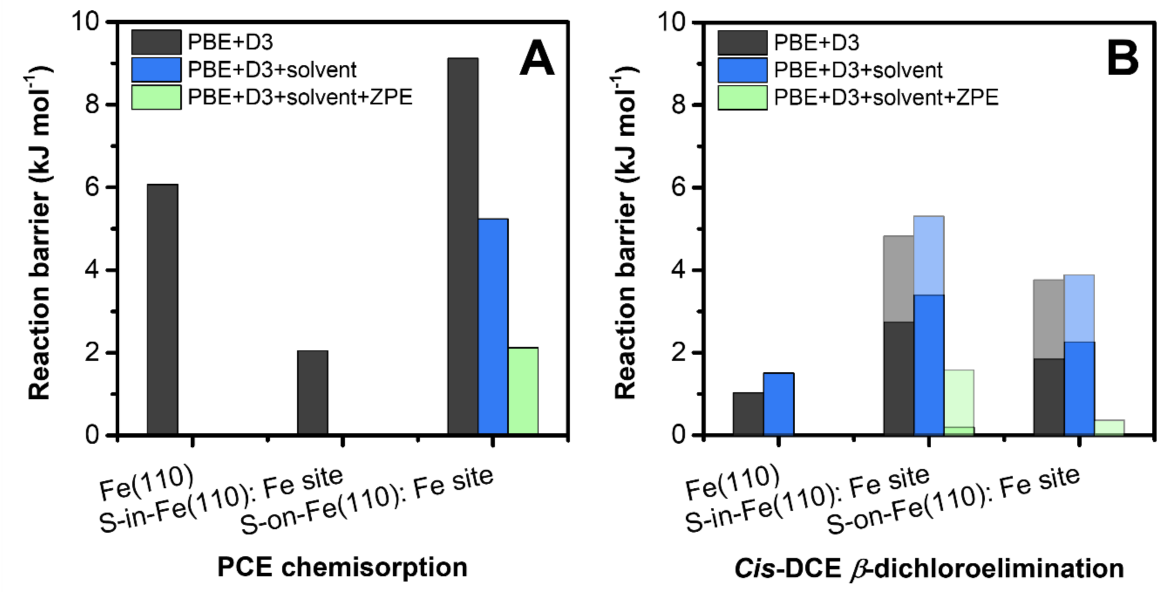


**Figure S14.** Reaction barriers corrected for implicit solvation and zero-point energy of (A) PCE chemisorption and (B) *cis*-DCE *β*-dichloroelimination at various Fe sites. The opaque and semi-transparent columns in (B) depict barriers for *cis*-DCE adsorbed with Cl atoms towards the Fe surface and the S atom, respectively.

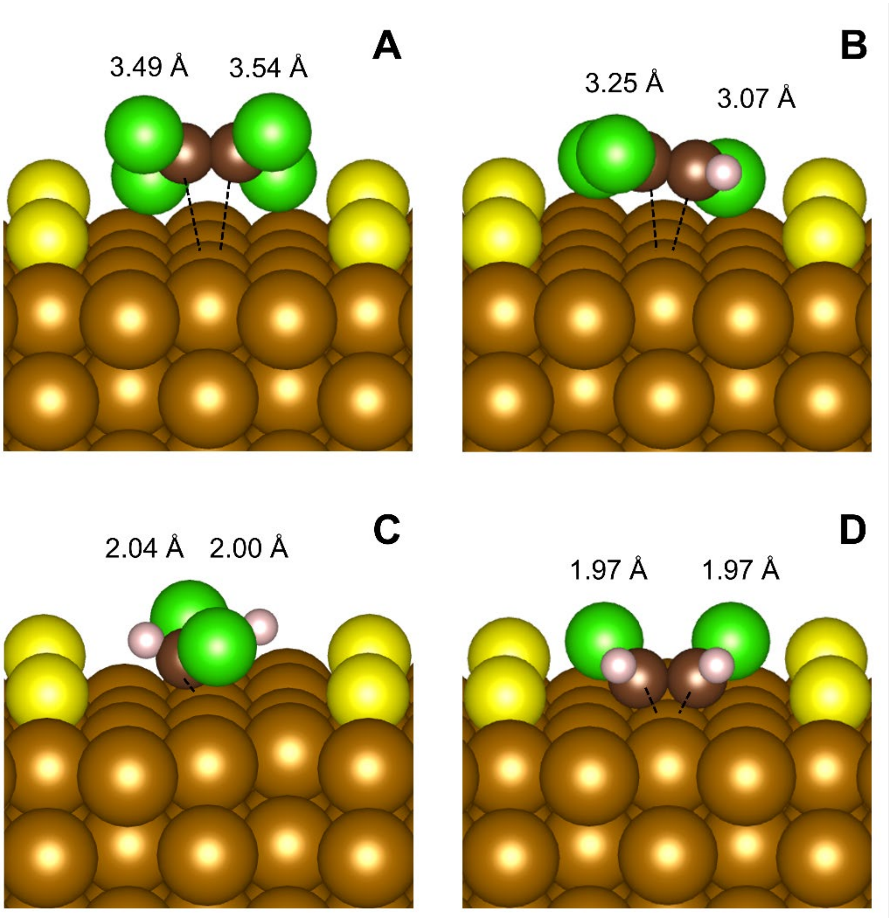


**Figure S15.** DFT+D3-optimized configurations of chlorinated ethenes adsorbed at the Fe site of the regularly sulfidated $S_{1/8\,ML}$-Fe(110) surface with shown C–Fe distances: (A) physisorbed PCE, (B) physisorbed TCE, (C) chemisorbed *trans*-DCE, and (D) chemisorbed *cis*-DCE. The adsorption complex in (C) was optimized with frozen C–Cl distances to prevent spontaneous Cl dissociation.

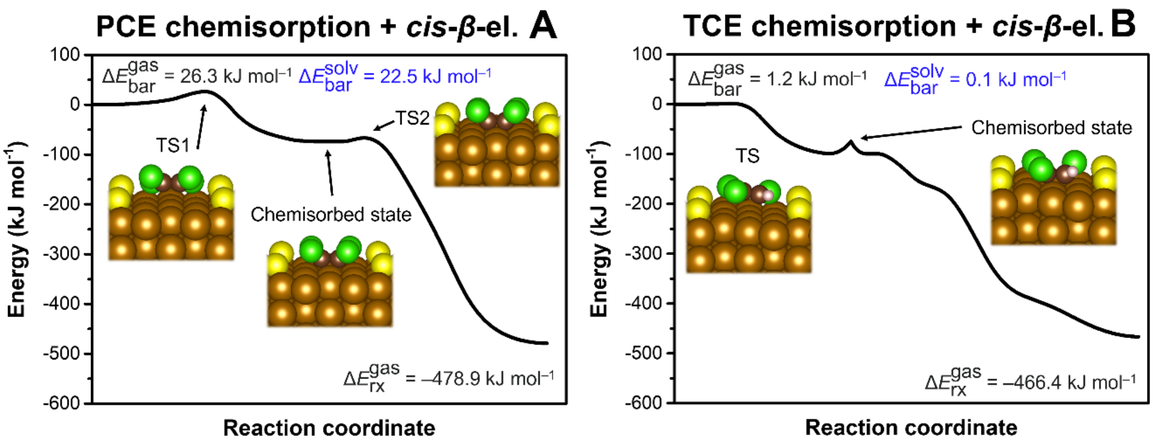


**Figure S16.** Reaction profiles of chemisorption and a consecutive *β*-dichloroelimination of (A) PCE and (B) TCE at the $S_{1/8\,ML}$-Fe(110) surface. CI-NEB calculations were performed in the gas phase (values in black). The solvent effect on the reaction barrier was included using a continuum solvation model with the structures of reactants and transition states taken from the CI-NEB calculation (value in blue). TS denotes the transition state. The chemisorbed state in (B) was calculated with fixed C–Cl distances to prevent spontaneous cleavage of Cl atoms during structural relaxations.

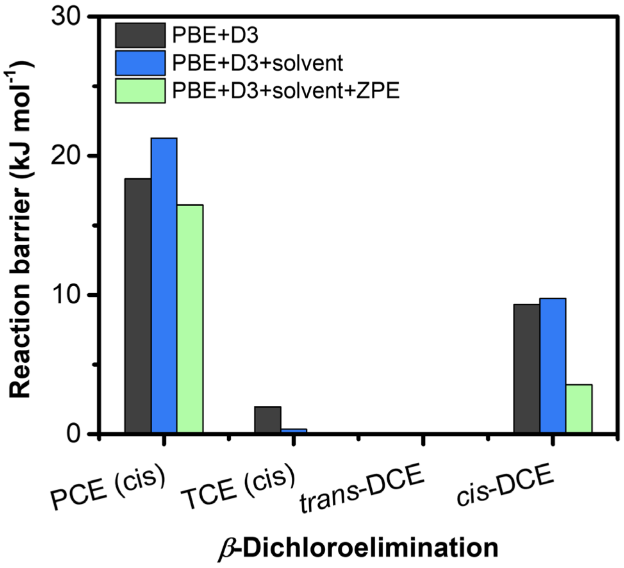


**Figure S17.** *β*-dichloroelimination barriers for chlorinated ethenes at the $S_{1/8\,ML}$-Fe(110) surface corrected for implicit solvation and zero-point energy.

**Table S3.** Horizontal displacement of S atoms in the $S_{1/8\,ML}$-Fe(110) surface model upon CE chemisorption. Atoms S1 and S2 were closer to the adsorbed CE molecules, as can be seen in Figures 4 and S18.

| | **Displacement of S atoms in the chemisorption complex (Å)** | | | | |
|---|---|---|---|---|---|
| **Molecule** | **S1** | **S2** | **S3** | **S4** | **Average** |
| PCE | 0.475 | 0.453 | 0.018 | 0.014 | 0.240 |
| TCE | 0.202 | 0.169 | 0.012 | 0.009 | 0.098 |
| *trans*-DCE | 0.087 | 0.041 | 0.015 | 0.006 | 0.037 |
| *cis*-DCE | 0.116 | 0.110 | 0.014 | 0.006 | 0.062 |

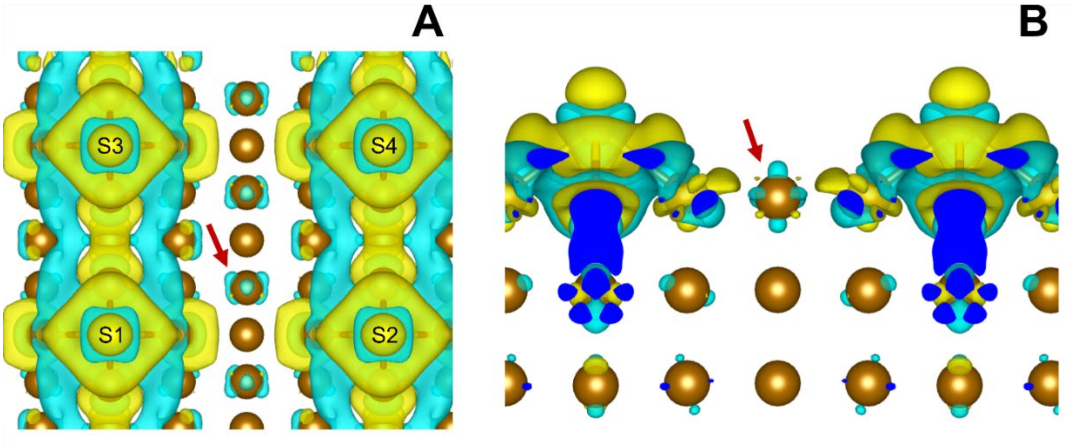


**Figure S18.** PBE-calculated charge density redistribution on the Fe(110) surface induced by four S adatoms: (A) top view, (B) side view. The yellow isosurface indicates an electron gain, while the blue one represents an electron loss. The isosurface level was set to 0.001 $Bohr^{-3}$. The CE adsorption site is indicated by a red arrow.

## References


(1) Henkelman, G.; Uberuaga, B. P.; Jónsson, H. A Climbing Image Nudged Elastic Band Method for Finding Saddle Points and Minimum Energy Paths. *J. Chem. Phys.* **2000**, *113* (22), 9901–9904. https://doi.org/10.1063/1.1329672.

(2) Brumovský, M.; Tunega, D. Intrinsic Effects of Sulfidation on the Reactivity of Zero-Valent Iron With Trichloroethene: A DFT Study. *J. Phys. Chem. C* **2023**, *127* (43), 21063–21074. https://doi.org/10.1021/acs.jpcc.3c04459.

(3) Grimme, S.; Ehrlich, S.; Goerigk, L. Effect of the Damping Function in Dispersion Corrected Density Functional Theory. *J. Comput. Chem.* **2011**, *32* (7), 1456–1465. https://doi.org/10.1002/jcc.21759.

(4) Grimme, S.; Antony, J.; Ehrlich, S.; Krieg, H. A Consistent and Accurate Ab Initio Parametrization of Density Functional Dispersion Correction (DFT-D) for the 94 Elements H-Pu. *J. Chem. Phys.* **2010**, *132* (15), 154104. https://doi.org/10.1063/1.3382344.

(5) Sun, J.; Remsing, R. C.; Zhang, Y.; Sun, Z.; Ruzsinszky, A.; Peng, H.; Yang, Z.; Paul, A.; Waghmare, U.; Wu, X.; et al. Accurate First-Principles Structures and Energies of Diversely Bonded Systems from an Efficient Density Functional. *Nat. Chem.* **2016**, *8* (9), 831–836. https://doi.org/10.1038/nchem.2535.

(6) Momma, K.; Izumi, F. VESTA 3 for Three-Dimensional Visualization of Crystal, Volumetric and Morphology Data. *J. Appl. Crystallogr.* **2011**, *44* (6), 1272–1276. https://doi.org/10.1107/S0021889811038970.

(7) Kisiel, Z.; Pszczółkowski, L. Assignment and Analysis of the Mm-Wave Rotational Spectrum of Trichloroethylene: Observation of a New, ExtendedbR-Band and an Overview of High-J,R-Type Bands. *J. Mol. Spectrosc.* **1996**, *178* (1), 125–137. https://doi.org/10.1006/jmsp.1996.0165.

(8) Tang, W.; Sanville, E.; Henkelman, G. A Grid-Based Bader Analysis Algorithm without Lattice Bias. *J. Phys. Condens. Matter* **2009**, *21* (8), 084204. https://doi.org/10.1088/0953-8984/21/8/084204.

(9) Yu, M.; Trinkle, D. R. Accurate and Efficient Algorithm for Bader Charge Integration. *J. Chem. Phys.* **2011**, *134* (6). https://doi.org/10.1063/1.3553716.

(10) Blöchl, P. E.; Jepsen, O.; Andersen, O. K. Improved Tetrahedron Method for Brillouin-Zone Integrations. *Phys. Rev. B* **1994**, *49* (23), 16223–16233. https://doi.org/10.1103/PhysRevB.49.16223.

(11) Wang, V.; Xu, N.; Liu, J.-C.; Tang, G.; Geng, W.-T. VASPKIT: A User-Friendly Interface Facilitating High-Throughput Computing and Analysis Using VASP Code. *Comput. Phys. Commun.* **2021**, *267*, 108033. https://doi.org/10.1016/j.cpc.2021.108033.

(12) Brumovský, M.; Micić, V.; Oborná, J.; Filip, J.; Hofmann, T.; Tunega, D. Iron Nitride Nanoparticles for Rapid Dechlorination of Mixed Chlorinated Ethene Contamination. *J. Hazard. Mater.* **2023**, *442*, 129988. https://doi.org/10.1016/j.jhazmat.2022.129988.

(13) Totten, L. A.; Roberts, A. L. Calculated One- and Two-Electron Reduction Potentials and Related Molecular Descriptors for Reduction of Alkyl and Vinyl Halides in Water. *Crit. Rev. Environ. Sci. Technol.* **2001**, *31* (2), 175–221. https://doi.org/10.1080/20016491089208.

(14) Khvostenko, O. G. Electronically Excited States of Chloroethylenes: Experiment and DFT Calculations in Comparison. *J. Electron Spectros. Relat. Phenomena* **2014**, *195*, 220–229. https://doi.org/10.1016/j.elspec.2014.07.015.
